# Predictive values derived from lower wisdom teeth developmental stages on orthopantomograms to calculate the chronological age in adolescence and young adults as a prerequisite to obtain age-adjusted informed patient consent prior to elective surgical procedures in young patients with incomplete or mismatched personal data

**DOI:** 10.3205/iprs000102

**Published:** 2016-12-06

**Authors:** Reinhard E. Friedrich, Kirsten Schmidt, András Treszl, Jan F. Kersten

**Affiliations:** 1 Klinik für Mund-, Kiefer- und Gesichtschirurgie, Kopf- und Neuro-Zentrum, Universitätsklinikum Hamburg-Eppendorf, Hamburg, Deutschland; 2 Institut für Medizinische Biometrie und Epidemiologie, Zentrum für Experimentelle Medizin, Universitätsklinikum Hamburg-Eppendorf, Hamburg, Deutschland; 3 Competenzzentrum Epidemiologie und Versorgungsforschung bei Pflegeberufen (CVcare), Institut für Versorgungsforschung in der Dermatologie und bei Pflegeberufen (IVDP), Universitätsklinikum Hamburg-Eppendorf (UKE), Hamburg, Deutschland

**Keywords:** informed consent to medical treatment, biometry, age determination by teeth, wisdom tooth development, forensic odontology

## Abstract

**Introduction:** Surgical procedures require informed patient consent, which is mandatory prior to any procedure. These requirements apply in particular to elective surgical procedures. The communication with the patient about the procedure has to be comprehensive and based on mutual understanding. Furthermore, the informed consent has to take into account whether a patient is of legal age. As a result of large-scale migration, there are eventually patients planned for medical procedures, whose chronological age can’t be assessed reliably by physical inspection alone. Age determination based on assessing wisdom tooth development stages can be used to help determining whether individuals involved in medical procedures are of legal age, i.e., responsible and accountable. At present, the assessment of wisdom tooth developmental stages barely allows a crude estimate of an individual’s age. This study explores possibilities for more precise predictions of the age of individuals with emphasis on the legal age threshold of 18 years.

**Material and Methods:** 1,900 dental orthopantomograms (female 938, male 962, age: 15–24 years), taken between the years 2000 and 2013 for diagnosis and treatment of diseases of the jaws, were evaluated. 1,895 orthopantomograms (female 935, male 960) of 1,804 patients (female 872, male 932) met the inclusion criteria. The archives of the Department of Diagnostic Radiology in Dentistry, University Medical Center Hamburg-Eppendorf, and of an oral and maxillofacial office in Rostock, Germany, were used to collect a sufficient number of radiographs. An effort was made to achieve almost equal distribution of age categories in this study group; ‘age’ was given on a particular day. The radiological criteria of lower third molar investigation were: presence and extension of periodontal space, alveolar bone loss, emergence of tooth, and stage of tooth mineralization (according to Demirjian). Univariate and multivariate general linear models were calculated. Using hierarchical multivariate analyses a formula was derived quantifying the development of the four parameters of wisdom tooth over time. This model took repeated measurements of the same persons into account and is only applicable when a person is assessed a second time. The second approach investigates a linear regression model in order to predict the age. In a third approach, a classification and regression tree (CART) was developed to derive cut-off values for the four parameters, resulting in a classification with estimates for sensitivity and specificity.

**Results:** No statistically significant differences were found between parameters related to wisdom tooth localization (right or left side). In univariate analyses being of legal age was associated with consecutive stages of wisdom tooth development, the obliteration of the periodontal space, and tooth emergence, as well with alveolar bone loss; no association was found with tooth mineralization. Multivariate models without repeated measurements revealed imprecise estimates because of the unknown individual-related variability. The precision of these models is thus not very good, although it improves with advancing age. When calculating a CART-analysis and a receiver operating characteristics – area under the curve of 78% was achieved; when maximizing both specificity and sensitivity, a Youden’s index of 47% was achieved (with 73% specificity and 74% sensitivity).

**Discussion:** This study provides a basis to help determine whether a person is 18 years or older in individuals who are assumed to be between 15 and 24 years old. From repeated measurements, we found a linear effect of age on the four parameters in the individuals. However, this information can't be used for prognosis, because of the large intra-individual variability. Thus, although the development of the four parameters can be estimated over time, a direct conclusion with regard to age can’t be drawn from the parameters without previous biographic information about a person. While a single parameter is of limited value for calculating the target age of 18 years, combining several findings, that can be determined on a standard radiography, may potentially be a more reliable diagnostic tool for estimating the target age in both sexes. However, a high degree of precision can’t be achieved. The reason for persistent uncertainty lies in the wide chronological range of wisdom tooth development, which stretches from well below to above the 18^th^ life year. The regression approach thus seems not optimal. Although sensitivity and specificity of the CART-model are moderately high, this model is still not reliable as a diagnostic tool. Our findings could have impact, e.g. on elective surgeries for young individuals with unknown biography. However, these results cannot replace social engagement, in particular thorough physical examination of patients and careful registration of their histories. Further studies on the use of this calculation method in different ethnic groups would be desirable.

## Zusammenfassung

**Einleitung:** Die Durchführung operativer Eingriffe bedarf der rechtsgültigen Aufklärung. Diese hat insbesondere bei geplanten elektiven Eingriffen umfassend und verständlich zu erfolgen. Darüber hinaus hat sich die ärztliche Aufklärung hinsichtlich der Einwilligungserklärung an der juristisch definierten Volljährigkeit zu orientieren. Infolge der großen transnationalen Migrationen gibt es einen Anteil an Patienten, deren Lebensalter unbekannt ist und deren ärztliche Alterseinschätzung das Erreichen des 18. Lebensjahres ausschließlich anhand des äußeren Erscheinungsbildes nur unsicher vermuten lässt. In dieser Situation kann die aus der forensischen Odontostomatologie bekannte Altersschätzung nach den radiologisch bestimmten Entwicklungsphasen der Weisheitszahnentwicklung die juristisch relevante Entscheidung unterstützen, ob die ärztliche Aufklärung einem Heranwachsenden oder Erwachsenen gilt. Da die Beurteilung der Zahnwurzelentwicklungsstadien der Weisheitszähne lediglich einen Zeitkorridor des wahrscheinlichen Lebensalters vorgibt, war es das Anliegen dieser Untersuchung, mit zusätzlichen Parametern der Radiomorphologie des unteren Weisheitszahns die Altersschätzung für das Erreichen des 18. Lebensjahres zu präzisieren.

**Material und Methoden:** Es wurden 1.900 Orthopantomogramme der Kiefer von Patienten mit bekanntem Geburtsdatum untersucht, 5 Orthopantomogramme entsprachen nicht den Einschlusskriterien. Das Lebensalter lag zwischen 15 und 24 Jahren. Es wurde eine Gleichverteilung der Untersuchungsgruppe nach Alter (je Lebensjahr) und Geschlecht bei der Auswahl der Röntgenbilder angestrebt und hinreichend eingehalten. Die Röntgenaufnahmen wurden in den Jahren 2000 bis 2013 in der Abteilung Zahnärztliche Radiologie des Universitätsklinikums Hamburg-Eppendorf oder in einer mund-kiefer-gesichtschirurgischen Praxis in Rostock angefertigt. Eine mehrfache Untersuchung von Patienten (n=1.804, mit 1.895 auswertbaren Orthopantomogrammen) war nicht ausgeschlossen und wurde ggf. bei den Analysen berücksichtigt. An den unteren Weisheitszähnen wurden vier Parameter bestimmt: Nachweis und Ausdehnung des Parodontalspaltes bzw. der periradikulären Sklerose, vertikaler Knochenverlust des Alveolarfortsatzes im Bereich des Zahnes, Durchbruchsstadium des Zahnes in die Mundhöhle und das Wurzelentwicklungsstadium (nach Demirjian). Nach einer Überprüfung, ob bereits die Erhebung eines Parameters ausreicht und hinreichend gute Erkenntnisse über die Volljährigkeit des Untersuchten liefert, wurden die erhobenen Parameter in drei Ansätzen kombiniert; hier wurde das Augenmerk auf unterschiedliche Aspekte, Anwendungen und Aussagen gerichtet. Die erste Analyse betrachtet die Entwicklung der vier Parameter im jeweiligen Patienten über die Zeit. Der zweite Ansatz untersucht die Vorhersage des Lebensalters basierend auf allen vier erhobenen Parametern unter Berücksichtigung der Messwiederholungen mittels eines gemischten linearen Modells, und schließlich wurden ein Gesamtmodell und geschlechtsspezifische Entscheidungsbäume erstellt, mit deren Hilfe bei Vorliegen der vier erhobenen Parameter eine Abschätzung der Wahrscheinlichkeit für das Erreichen bzw. Nicht-Erreichen der Volljährigkeit abgeleitet werden kann.

**Ergebnisse:** Es ließen sich keine signifikanten Unterschiede hinsichtlich der Lokalisation der Weisheitszähne (rechts/links) nachweisen. In den univariaten ROC-AUC-Analysen zeigten die Parameter Zahndurchbruchsstadium, Parodontalspalt und der Knochenschwund des den Weisheitszahn umgebenden Alveolarfortsatzes einen klaren signifikanten prognostischen Wert hinsichtlich der Volljährigkeit in der Untersuchungsgruppe. Das Mineralisationsstadium zeigte sich nicht als geeigneter Parameter zur direkten Bestimmung der Volljährigkeit. Für die Prognose scheinen drei der vier Parameter somit geeignet. Betrachtet man die Entwicklung der Zähne in Individuen anhand der vier Parameter mit wachsendem Lebensalter, konnte eine Veränderung der vier Zahnparameter nachgewiesen werden. Eine Umkehr der Kausalität und die Vorhersage des Lebensalters durch die vier Zahnparameter erscheint jedoch problematisch. Eine erhebliche individuelle Komponente ist bei den untersuchten Personen zu beobachten. Dies liegt u.a. daran, dass eine Entwicklung der Weisheitszähne durchaus vor und nach dem Erreichen des 18. Lebensjahres stattfinden kann. In multivariaten Analysen konnte gezeigt werden, dass durch die Kombination der Parameter mit wesentlich größerer Wahrscheinlichkeit ein Lebensalter von 18 Jahren und höher korrekt vorausgesagt werden kann und somit eine Formel die Kalkulation des Alters ermöglicht. Eine Trennung zwischen Heranwachsenden und Volljährigen ist mit einer Formel jedoch bei weitem nicht so perfekt, wie man es sich eigentlich wünschen würde. Ein Entscheidungsbaum, der nach geeigneten, aufeinander abgestimmten Cut-off-Werten sucht, verspricht eine leicht handhabbare Lösung zu liefern. Der Gesamtentscheidungsbaum hatte eine Fläche unter der Kurve (AUC) von 78%. Die Wahl der Entscheidungsregel setzt jedoch eine Abwägung der Priorisierung von Sensitivität oder Spezifität voraus. Ausgehend von der Annahme, dass beide Kriterien gleichwertig sind, lässt sich eine angestrebte Maximierung der Summe der beiden Maßzahlen anstreben. Die Ableitung eines Entscheidungsbaumes führte bei geeigneter Wahl der Knoten zu einer Sensitivität von 74% und einer Spezifität von 73% (hieraus folgt ein Youden-Index von 47%). Eine vollständig sichere Unterscheidung forensisch relevanter Lebensalter ist mit den vorgestellten Methoden somit nicht zu treffen.

**Diskussion:** In der ärztlichen Aufklärung ist besondere Sorgfalt auf die alters- und entwicklungsangepasste Kommunikation mit dem Patienten zu achten. Ein wesentlicher Pfeiler der juristischen Personeneinschätzung ist das Erreichen der Volljährigkeit, die in Deutschland im Grundsatz mit dem 18. Lebensjahr erreicht ist. Für Personen mit unbekanntem Geburtsdatum können gelegentlich Schwierigkeiten entstehen, auf welcher Grundlage die ärztliche Aufklärung zu erfolgen hat, wenn es sich dem Anschein nach um eine Person im fortgeschrittenen Heranwachsenden-Alter handelt. Unter der Voraussetzung, dass die ärztliche Einschätzung für einen geplanten Eingriff von einem einverständnisfähigen Patienten ausgeht, kann die hier vorgestellte Methode die Bestimmung des 18. Lebensjahres anhand einer radiologischen Standarduntersuchung der Kiefer mit Wahrscheinlichkeit leisten. Die biologische Breite der Weisheitszahnentwicklung liegt jedoch beidseits des 18ten Lebensjahres. Die Fehlerbreite nimmt mit zunehmendem Alter ab, insbesondere bei der Abschätzung der Volljährigkeit von tatsächlich bereits 21-Jährigen, erreicht aber auch hier keine vollständige Sicherheit der Abschätzung der Volljährigkeit. Die vorgelegten Untersuchungsergebnisse sind geeignet, die forensische Altersschätzung in mehreren Bereichen ärztlicher Tätigkeit fundiert zu unterstützen. Die Untersuchungsmethoden sind für den Behandler jedoch ungeeignet, anhand dieser Berechnungen sich der genauen Kenntnis der individuellen Lebensumstände des jeweiligen Patienten zu entziehen. Insbesondere die sorgfältig erhobene Lebensgeschichte und körperliche Untersuchung bleiben vorrangig zu bestimmende Werte der Personeneinschätzung. Die Erweiterung des Berechnungsansatzes durch Anwendung in anderen Populationen ist anzustreben.

## Introduction

**Figure 1:**
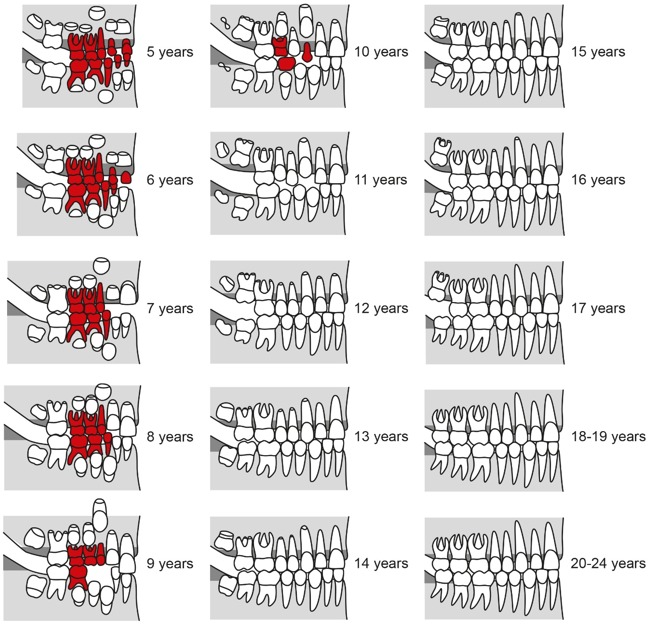
Development of the dentition in females, figure adapted from [43], slightly modified. Teeth of primary dentition are marked in red.

**Figure 2:**
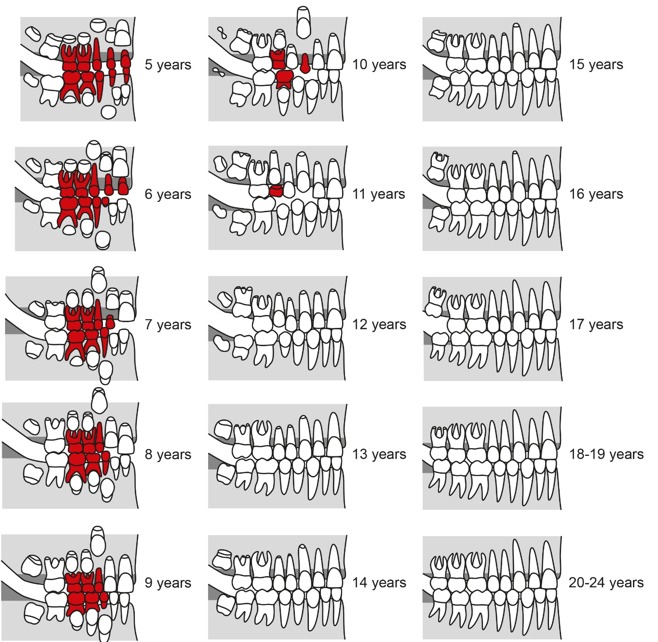
Development of the dentition in males adapted from [43], slightly modified. Teeth of primary dentition are marked in red.

The development of the human dentition is a long lasting process starting in certain phases of the embryo [100], continues in early childhood [39], [57], [59], [63], [43], [94] and will be completed passing distinct phases of tooth eruptions that can last up to the third decade of life [67], [84], [87] (Figure 1 (Fig. 1) and Figure 2 (Fig. 2)). The known association of certain phases of human dentition to chronological age was already used for different purposes, predominantly in dental treatment planning and in forensic sciences [1], [2], [4], [5], [7], [8], [9], [10], [11], [13], [14], [15], [16], [17], [18], [20], [21], [22], [23], [24], [25], [26], [27], [28], [30], [31], [32], [33], [34], [35], [36], [37], [39], [40], [41], [42], [46], [47], [48], [49], [50], [51], [52], [53], [54], [55], [56], [57], [58], [61], [62], [63], [65], [67], [68], [69], [70], [71], [72], [73], [74], [75], [76], [77], [78], [79], [80], [81], [82], [83], [85], [87], [88], [89], [90], [91], [94], [97], [99], [103], [104], [105], [106], [107], [108], [109], [110], [111], [112], [113]. The most prominent early usage of dental age determination was probably the oral investigation of children in England during the nineteenth century [87], [88] (quoted in [87]). In that time child labor was a common practice and children’s maturity to professional work was assessed by dental findings [87]. Identification and age assessment by means of tooth investigation is widely applied in the dental and forensic sciences in order to identify corpses and to estimate their likely age [53], [87]. With respect to forensic applications, dental, physical and radiographic findings are estimated relative to other findings of the skeleton [11], [12], [19], [27], [30], [44], [45], [52], [64], [86], [90], [91], [92], [93], [95], [101], [102], [108], [112], [113]. The application of age estimation based on dental findings in living individuals is a quite new development in the fields of forensic dentistry [61], anthropometry [104], and legal science [55]. The increased need for age estimation in living individuals is caused by an increase of border-crossing migration [3] and recent developments of child and youth crime [27]. In particular, refugees from belligerent countries will often be unable to clarify biometric data like date of birth. The uncertainty about the age of a patient can hamper the planning and implementation of medical procedures in the young patient [66], [91]. Given the capacity of individual judgement, even in children aged 12 to 18 years the right of self-determination has to be considered in the planning of medical procedures [29]. In surgical practice, the relevant age of a patient to agree in medical procedures is 18 years (§2 Bürgerliches Gesetzbuch, Bundesrepublik Deutschland). In particular, in planned elective surgical procedures of young patients with uncertain chronological age a technical aid would be valuable that allows the discrimination of the age of 18 years in order to provide a basis of informed patient consent in relation to age. 

**Figure 3:**
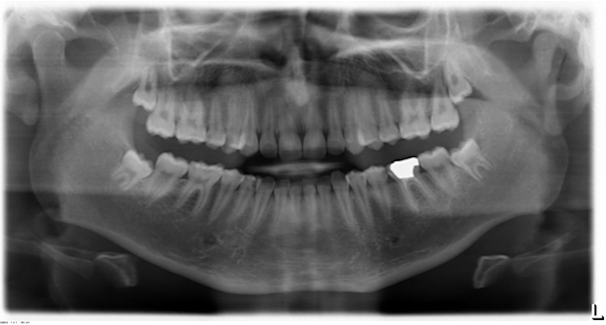
Orthopantomogram of the jaws and teeth. Lower third molars show incomplete root formation.

Recent studies provided some data allowing the estimation of a chronological age of 18 to 21 years based on the radiological determination of certain stages of wisdom tooth development [22], [31], [32], [33], [40], [41], [42], [43], [44], [45], [46], [47], [49] (Figure 3 (Fig. 3)). Other studies attempted to define the attainment of a chronological age of 18 years by analyzing other time-dependent biological or radiological findings of wisdom teeth with different methods [13], [14], [16], [17], [20], [21], [23], [34], [35], [56] and compared the quality of different age assessment methods [36], [52], [55], [72], [82], [83], [107]. The present study was designed to investigate several radiological findings measurable on wisdom teeth in order to more precisely determine a chronological age of 18 years or more in a given young individual.

## Material and methods

The study comprises the evaluation of orthopantomograms of patients taken between the years 2000 and 2013 for diagnosis and treatment of diseases of the jaws. The archives of the Department of Diagnostic Radiology in Dentistry, Eppendorf University Hospital, Hamburg, and of an oral and maxillofacial office in Rostock, Germany, were used to collect a sufficient number of radiographs. The study was approved by the local authority of the hospital as a prerequisite in the implementation of a medical dissertation in dentistry (K.S.). A total of 1,900 orthopantomograms were analyzed (females 938, males 962) aged 15 to 24 years, the complete case analysis included 1,895 orthopantomograms (females 935, males 960). Five patients did not meet inclusion criteria. Multiple radiographs performed at different times were evaluable in 85 patients. A special focus was laid on the almost equal distribution of age categories. The collection of X-ray images has been so far continued consecutively until the target size has been reached completely or at least approximately, i.e. 100 people per single age for the period of 15 to 24 year-old males and females. Age was rounded by year (Table 1 (Tab. 1)), e.g. a person was calculated as aged fifteen from the day of the 15^th^ birthday to the last day before the following birthday. However, for regression analyses, age was determined by number of days in order to estimate the parameters precisely. Patients with a history of trauma, neoplasia, or maxillofacial deformity were excluded from study (n=5).

**Figure 4:**
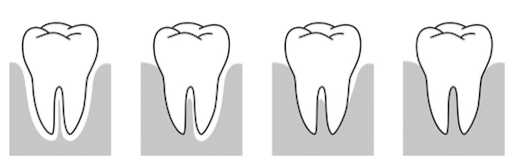
Staging of the third molar’s periodontal space’ visibility on orthopantomograms according to Olze et al. [75]. The stages are defined in Table 2.

**Figure 5:**
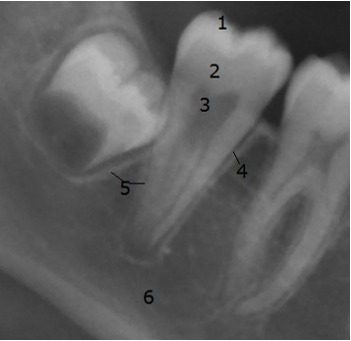
Cropped image of orthopantomogram showing lower molars of the right side. The third molar’s crown is radiologically of full radiopacity, the enamel to dentine border can be seen and roots are not developed: a continuous radiopaque line surrounds the developing tooth (1 = enamel, 2 = dentine, 3 = pulp cavity, 4 = periodontal space, 5 = lamina dura, 6 = cortical bone (lower border of mandible).

**Figure 6:**
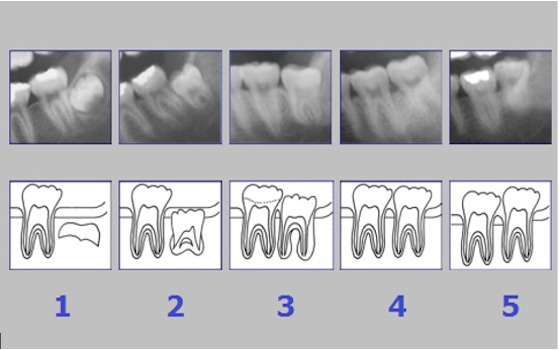
Stages of third molar eruption [72], [78]

**Figure 7:**
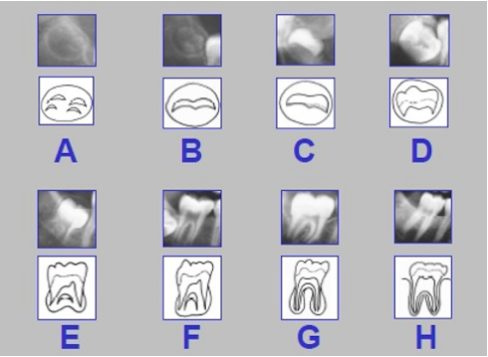
Stages of tooth/root mineralization [15]

The radiological criteria of lower third molar investigation were: presence and extension of periodontal space (Figure 4 (Fig. 4)), alveolar (periodontal) bone loss (Figure 5 (Fig. 5)), emergence of tooth (Figure 3 (Fig. 3), Figure 5 (Fig. 5) and Figure 6 (Fig. 6)), and stage of tooth mineralization (Figure 7 (Fig. 7)). Teeth were identified according to the dental scheme proposed by Féderation Dentaire International (FDI) including a consecutive numbering of both the teeth and the related quadrant of the jaws. The visibility of the periodontal space was staged according to proposals of Olze et al. [75] (Table 2 (Tab. 2)). In order to allow the evaluation of this item independent from the development stage of the tooth, this stage grouping was modified with respect to a certain radiological finding: the periodontal space is a fine radiotranslucent structure delineating the dental root from the surrounding bone. On radiographs, the jaw’s border to the periodontal space is frequently marked by a finely drawn radiopaque line entitled ‘lamina dura’ [24]. This radiological term can be applied to teeth with developing roots and also in the cases of completely mineralized teeth (Figure 7 (Fig. 7)). Alveolar bone loss was estimated according to Olze et al. [77] (Table 3 (Tab. 3)). 

Tooth eruption was categorized according to Olze et al. [78], (Table 4 (Tab. 4)). The root development and mineralization was classified according to Demirjian et al. [15] (Figure 7 (Fig. 7), Table 5 (Tab. 5)).

### Statistical methods

Statistical analyses were calculated for patients with complete cases for the relevant parameters that meet the inclusion criteria, i.e. otherwise healthy individuals with no history of trauma or skeletal dysplasia who had been investigated for their dental status and showed third molars on radiographs. Descriptive analysis is given by arithmetic mean, standard deviation, the three central quartiles – stratified by gender when appropriate. Multivariate models are reported with estimates, p-values and 95%-confidence intervals. For receiver operating characteristics (ROC) curves and hierarchical models we used information of both teeth, for the remaining analyses the information derived from radiographic findings of both teeth were combined as the arithmetic mean and constitute the basis for calculations of age determination.

We conducted three different approaches for estimation of age or ‘legal stage’ respectively: an hierarchical model which took all measurements of the individuals into account. 

A reliable estimate of the age of 18 years cannot be calculated with this combination of dental findings *alone*, as we can show in calibration models; here we adjusted an overall model with interactions as well as we stratified analysis by gender. To distinguish adults from adolescents a Classification And Regression Tree (CART) analysis was performed by applying an unbiased recursive partitioning algorithm in order to predict the status of legal age to identify optimal cut-off values for each variable; the selection of elected nodes results in a combination of sensitivity and specificity where the maximization of Youden’s index was presumed as desirable. Statistical software package R 3.2.3 was used for all calculations. P-values are reported without correction for multiple testing. Level of significance was set to p<0.05, two tailed.

## Results

Both univariate and multivariate analyses revealed no statistical significant differences of parameters related to the localization of wisdom tooth (right or left side) (Wilcoxon-Mann-Whitney-U test, mixed model). 

### 1. Evaluation of single parameters

#### 1.1 Periodontal space

**Figure 8:**
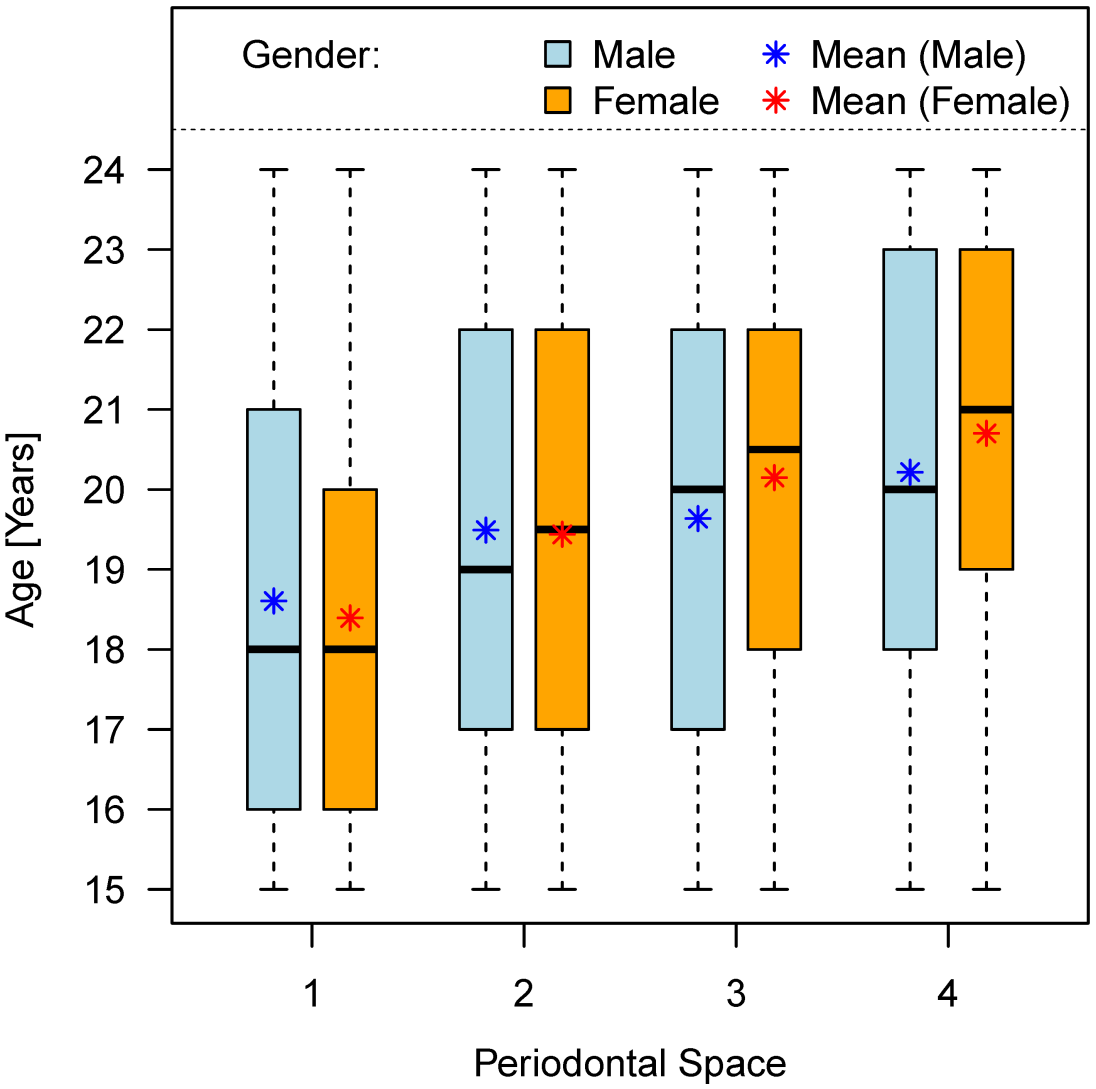
Chronological age and stage of periodontal space visibility (teeth No. 38 and 48)

The stages of periodontal space loss with respect to gender are summarized in Table 6 (Tab. 6) and shown in Figure 8 (Fig. 8).

#### 1.2 Alveolar bone loss

**Figure 9:**
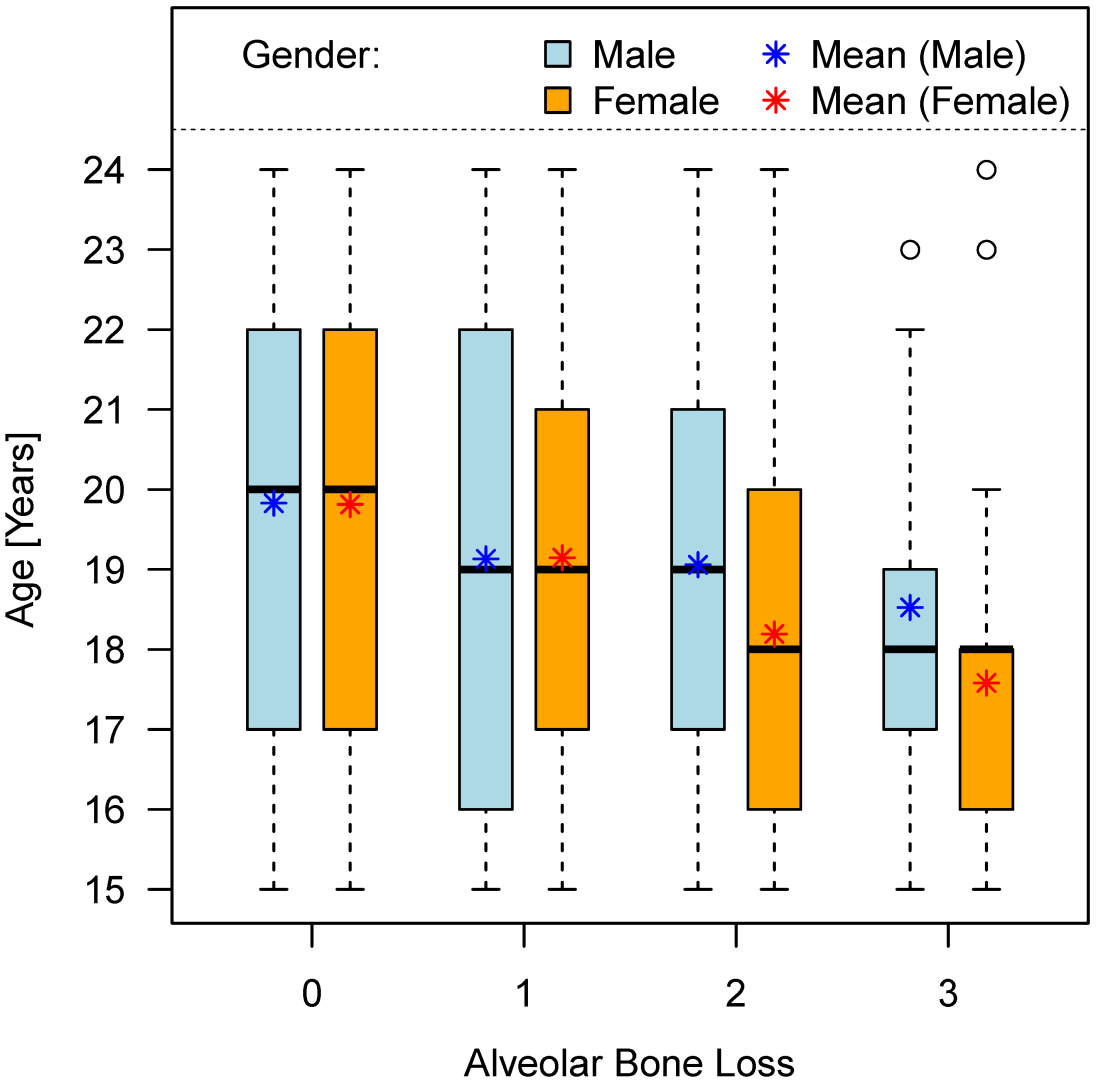
Chronological age and stage of alveolar (periodontal) bone loss (teeth No. 38 and 48)

Age distribution of bone loss was investigated but proved no meaningful results concerning a predictability of age of this age group depending on wisdom teeth’ alveolar bone height (Table 7 (Tab. 7)), (Figure 9 (Fig. 9)).

#### 1.3 Wisdom tooth emergence

**Figure 10:**
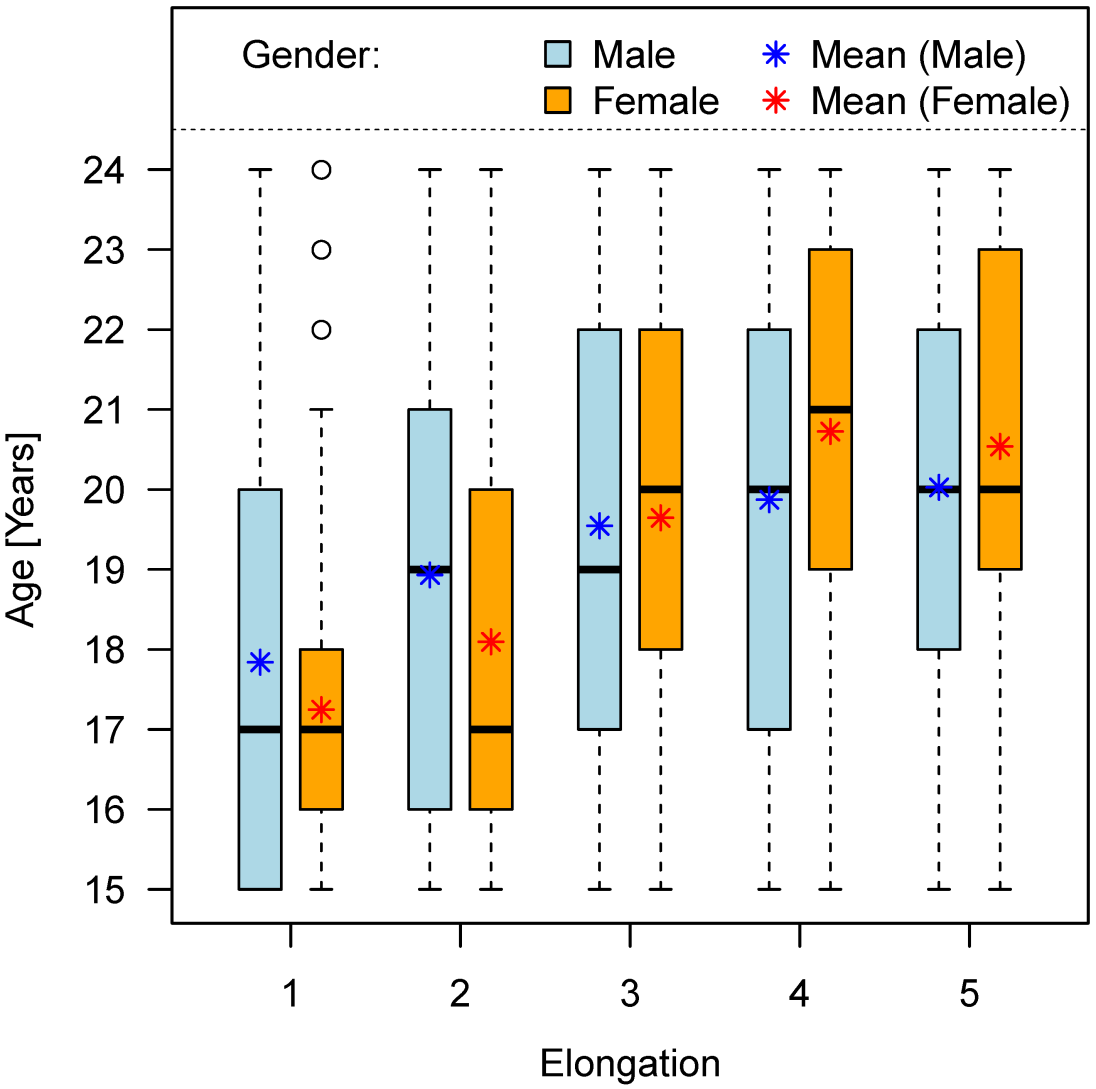
Chronological age and stage of wisdom tooth eruption/elongation (teeth No. 38 and 48)

Females are about 0.7 years ahead in the development stages 1 and 2 as compared to males. On the other hand, females showed 0.5 years developmental delay in stages 3, 4 and 5 compared to male. Fifty percent of males with wisdom tooth grown into occlusion were aged 20 years or more. This 50% value was reached by women aged at least 21 years. Mean age in individuals with the emergence stage 5, i.e. elongation of the lower wisdom tooth, was 20 years in males and 20.5 years in females. The results are summarized in Table 8 (Tab. 8) and shown in Figure 10 (Fig. 10).

#### 1.4 Development of dental root mineralization

**Figure 11:**
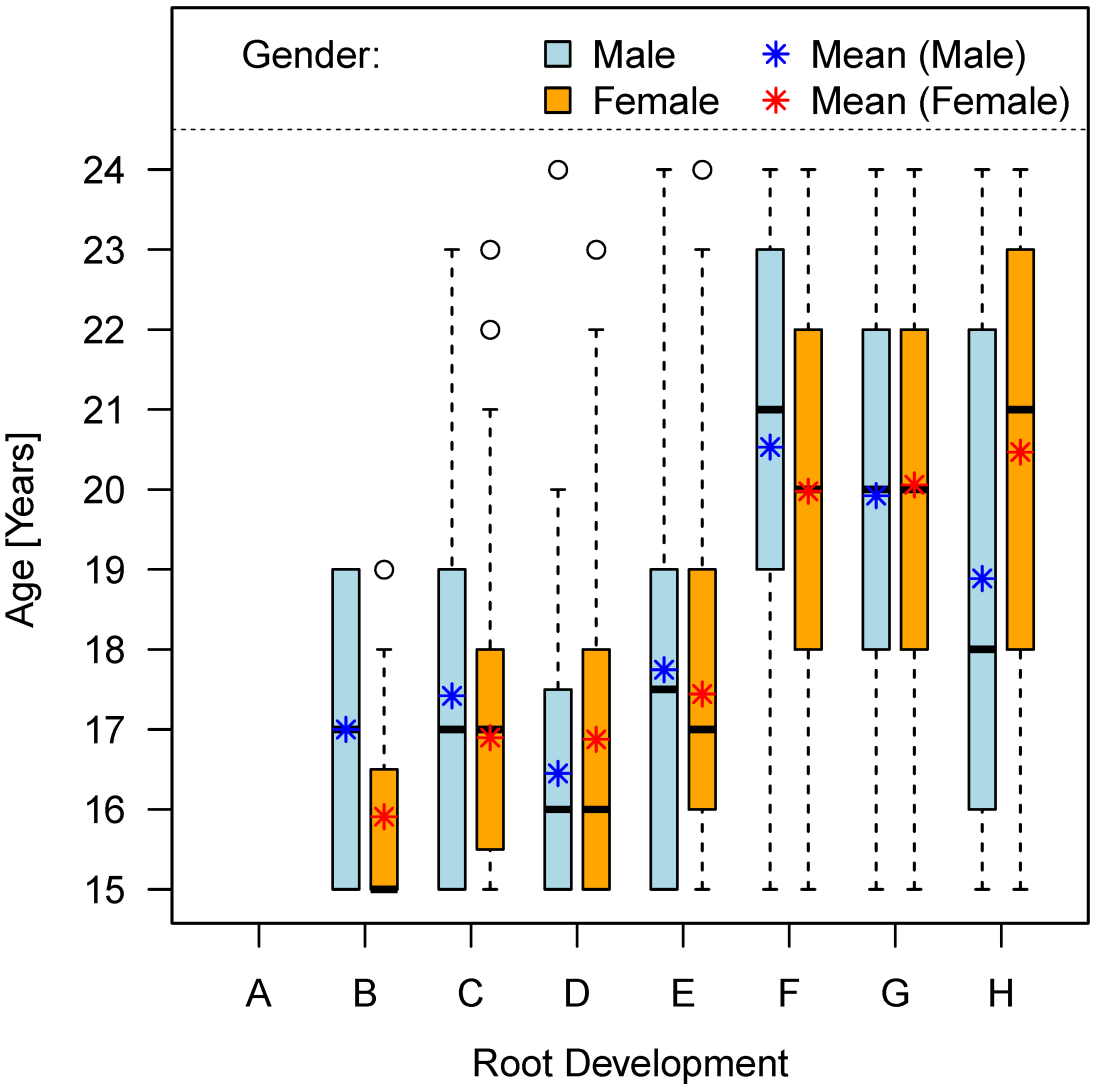
Chronological age and stage of root development (mineralization), (teeth No. 38 and 48)

Females are about 0.6 years ahead in the majority of development stages as compared to males. However, completion of root development (stage H) is about 1.5 years earlier on average in males compared to females (Table 9 (Tab. 9), Figure 11 (Fig. 11)). This result is in contradiction to current doctrine (Figure 1 (Fig. 1) and Figure 2 (Fig. 2)). 

**Figure 12:**
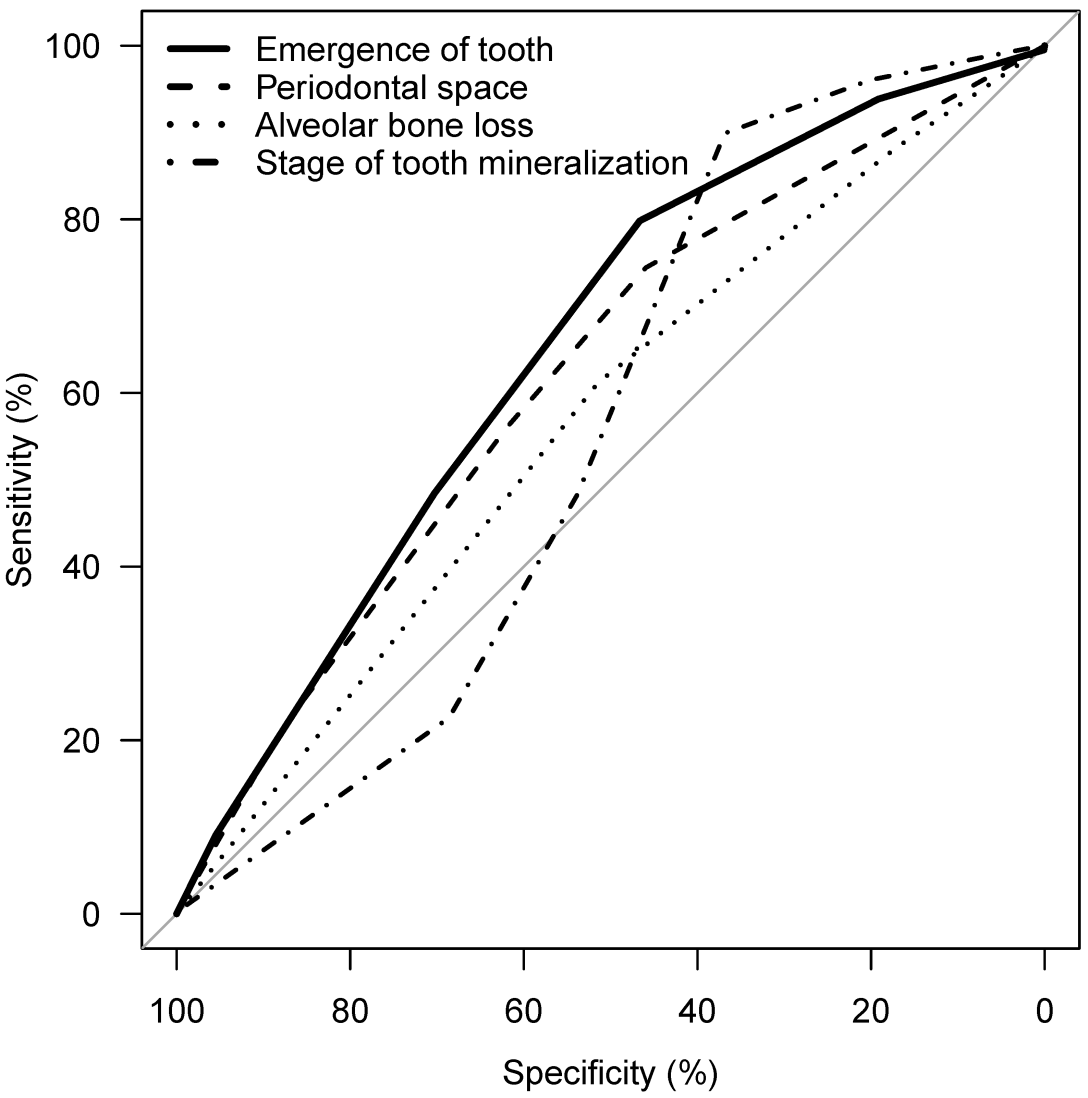
ROC curve of emergence of tooth, periodontal space visibility, alveolar bone loss, stage of tooth mineralization, target: attainment of majority (18 years of age), teeth 38 and 48

**Figure 13:**
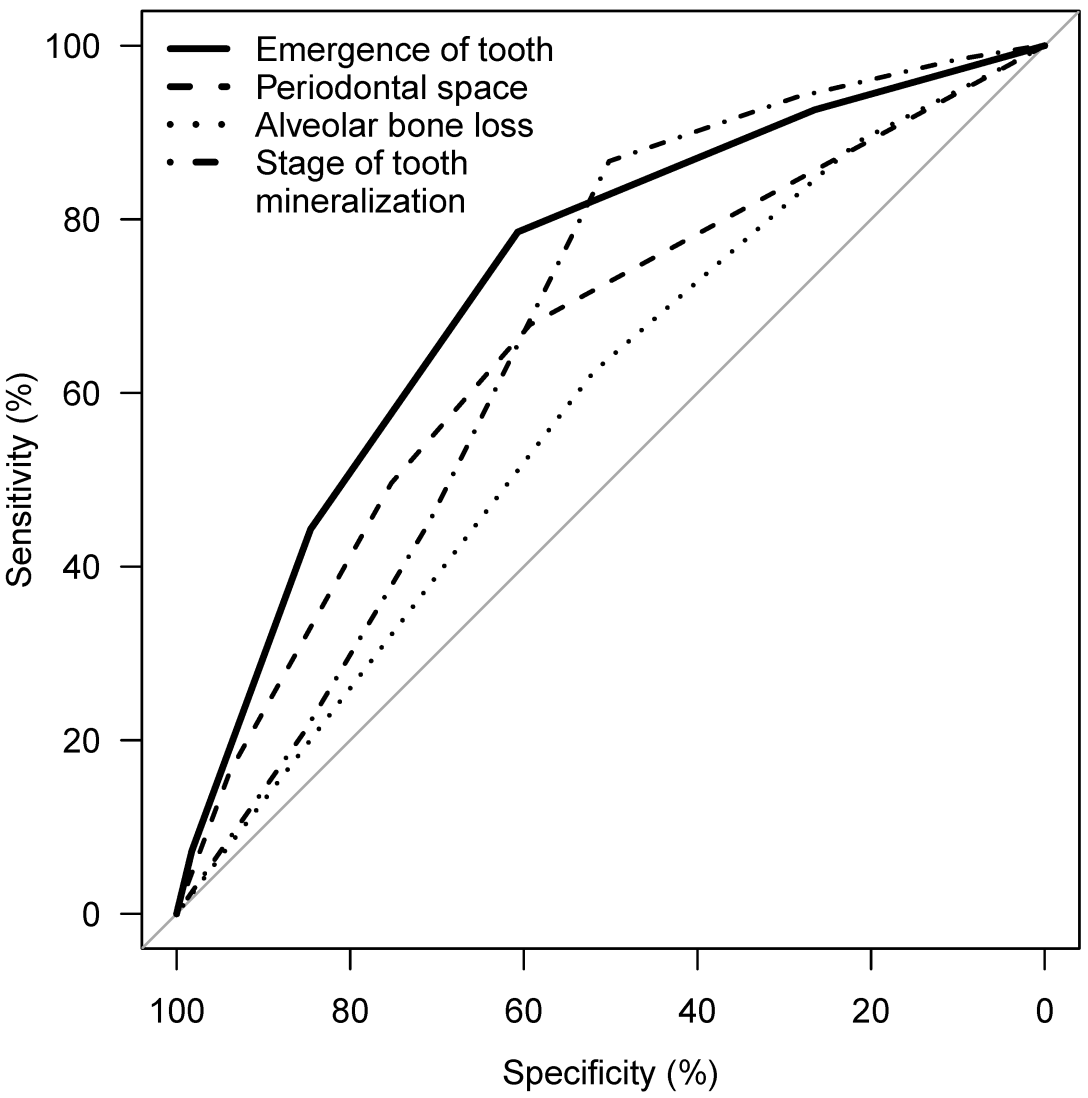
ROC curve of emergence of tooth, periodontal space visibility, alveolar bone loss, stage of tooth mineralization, target: attainment of majority (18 years of age), teeth 38 and 48 – females

**Figure 14:**
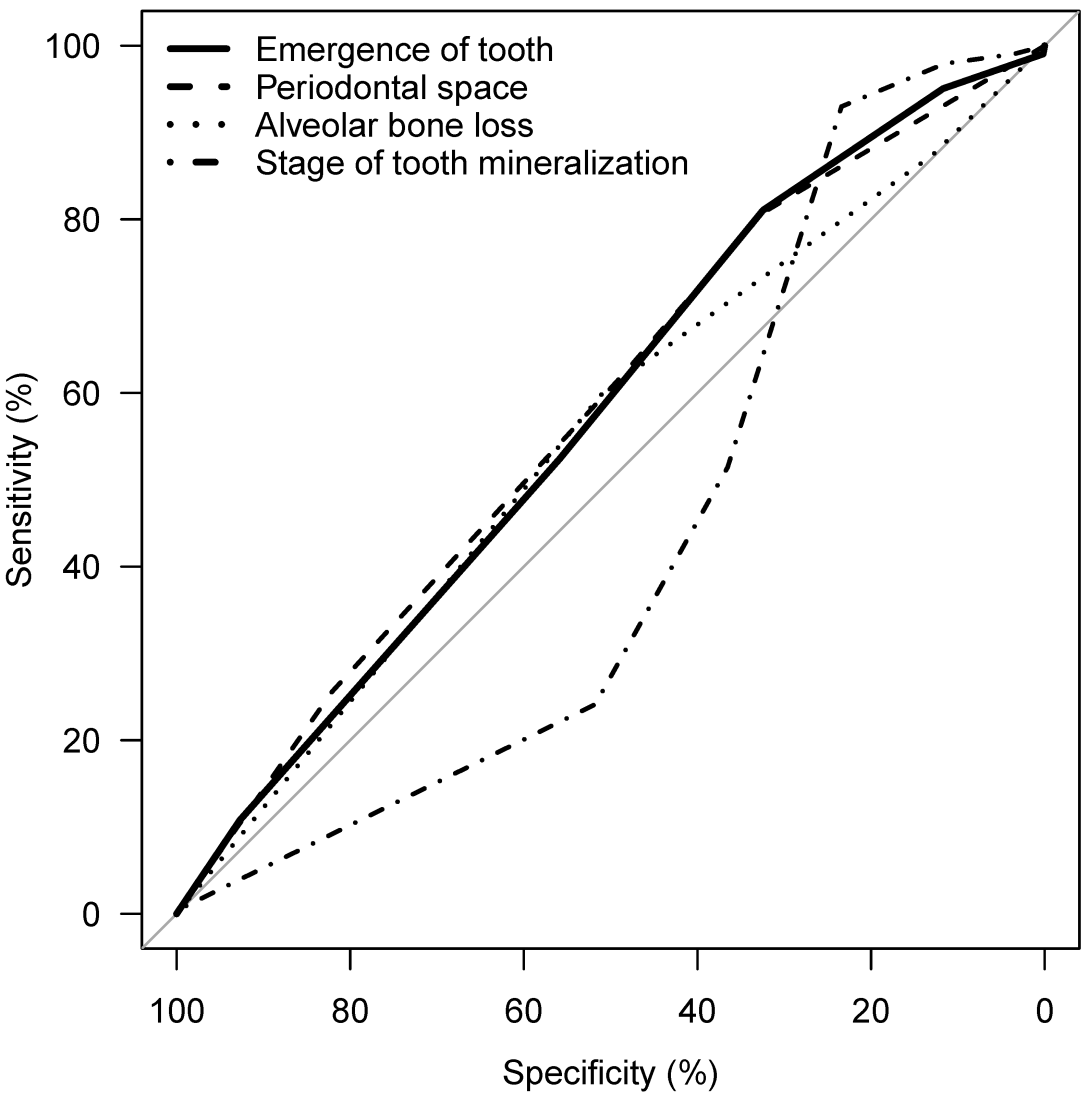
ROC curve of emergence of tooth, periodontal space visibility, alveolar bone loss, stage of tooth mineralization, target: attainment of majority (18 years of age), teeth 38 and 48 – males

Considering the parameters 1 to 4, an association of the applied stages and chronological age is apparent, with the exception of tooth mineralization (Figure 12 (Fig. 12)). The statistical measures of ROC curves are listed in Table 10 (Tab. 10). The ROC curves were calculated based on all orthopantomograms to determine the binary target level of attained majority (18 years of age or older) irrespective of gender. The AUC of each of the four parameters differ significantly from the value expected by chance (Table 10 (Tab. 10)). Further calculations were aimed to establish predictive table using models with respect to the individual parameters with respect to gender (Figure 13 (Fig. 13) and Figure 14 (Fig. 14)).

**Figure 15:**
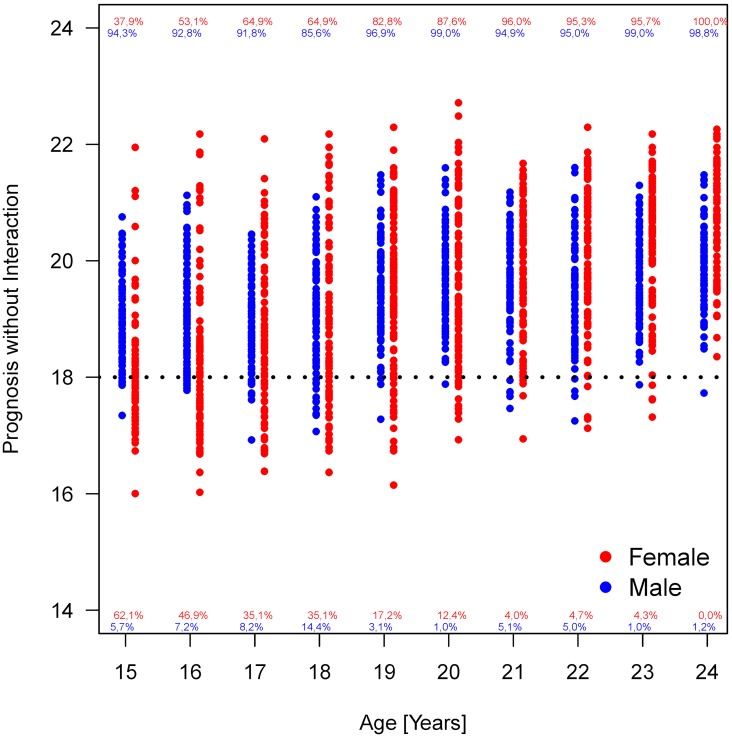
Relation between projected age and true age according to mixed model calculation without considering interactions of parameters. Percentages indicate the number of individuals per age group (year) calculated to be younger or older than 18 years old.

**Figure 16:**
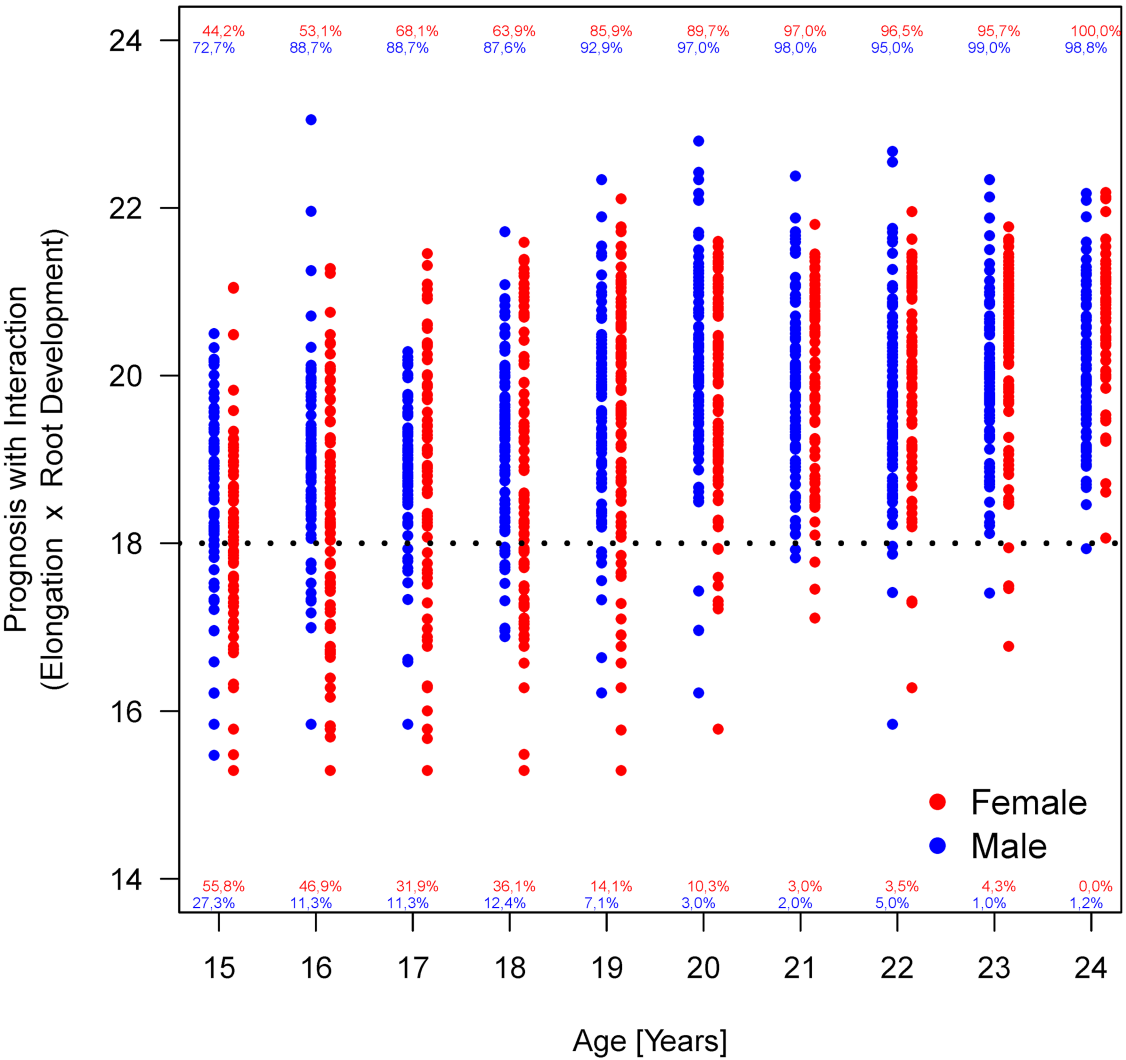
Relation between projected age and true age according to mixed model calculation considering interactions of parameters. Percentages indicate the number of individuals per age group (year) calculated to be younger or older than 18 years old.

**Figure 17:**
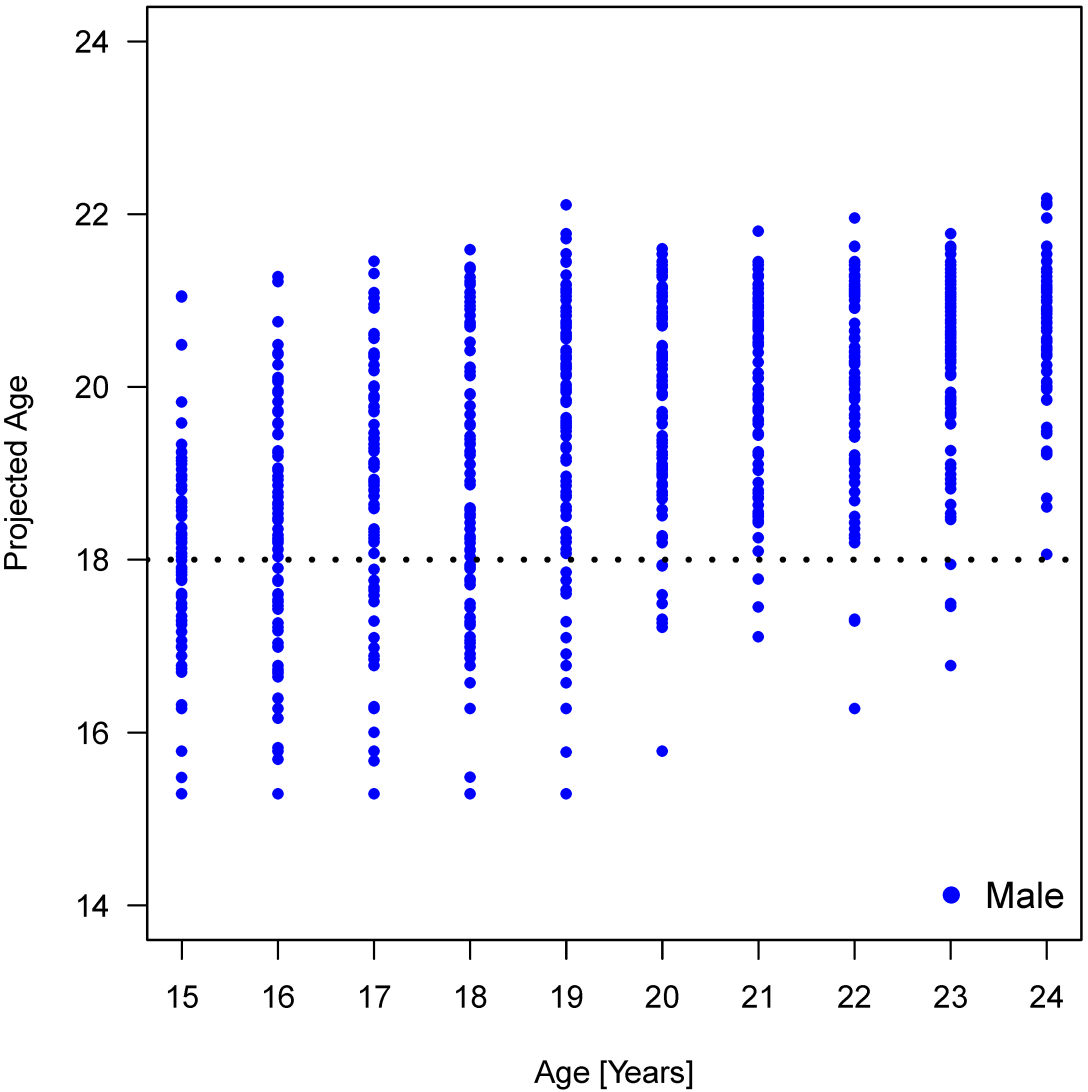
Relation between projected age and true age according to linear regression model calculation considering interactions of parameters elongation and root development: males

**Figure 18:**
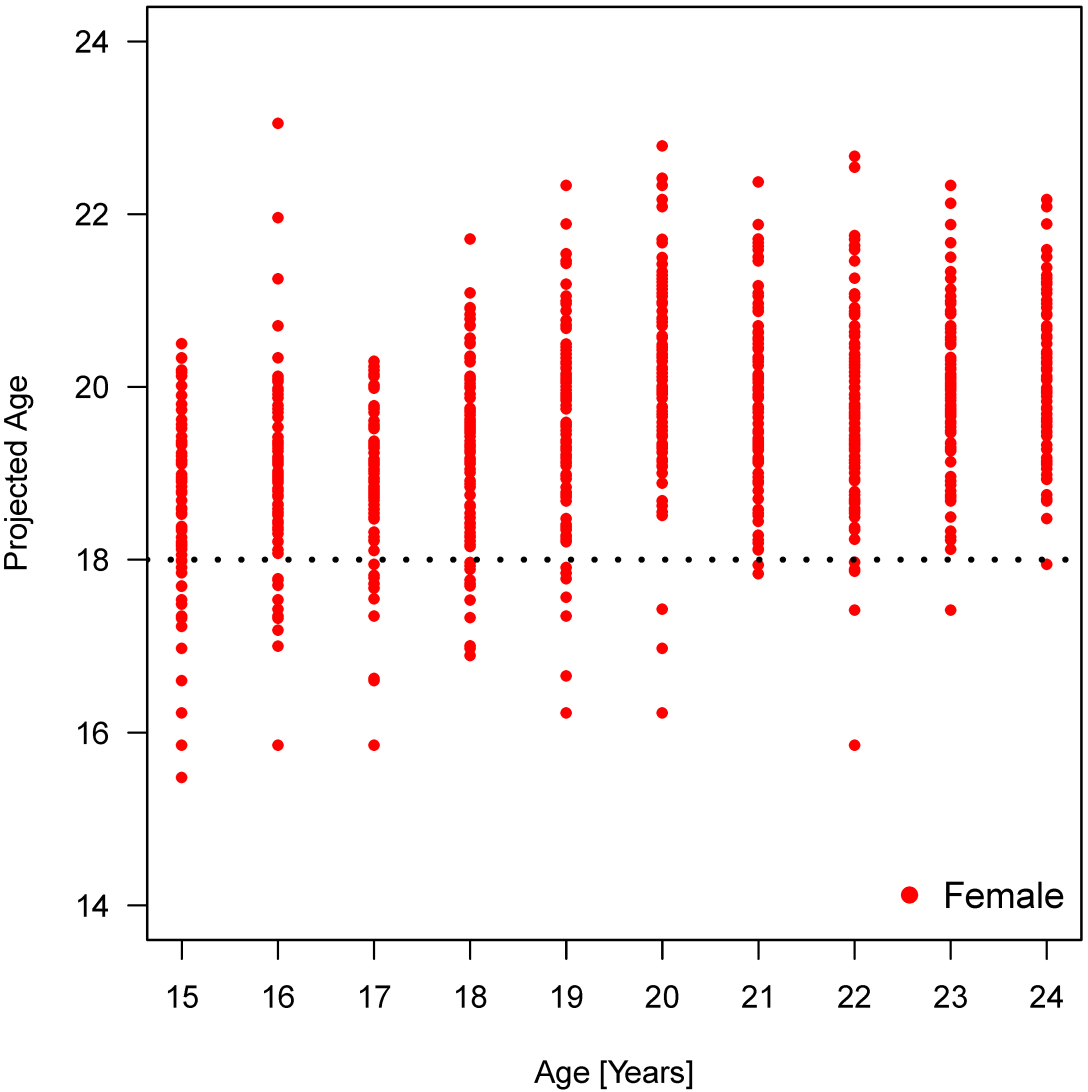
Relation between projected age and true age according to linear regression model calculation considering interactions of parameters elongation and root development: females

**Figure 19:**
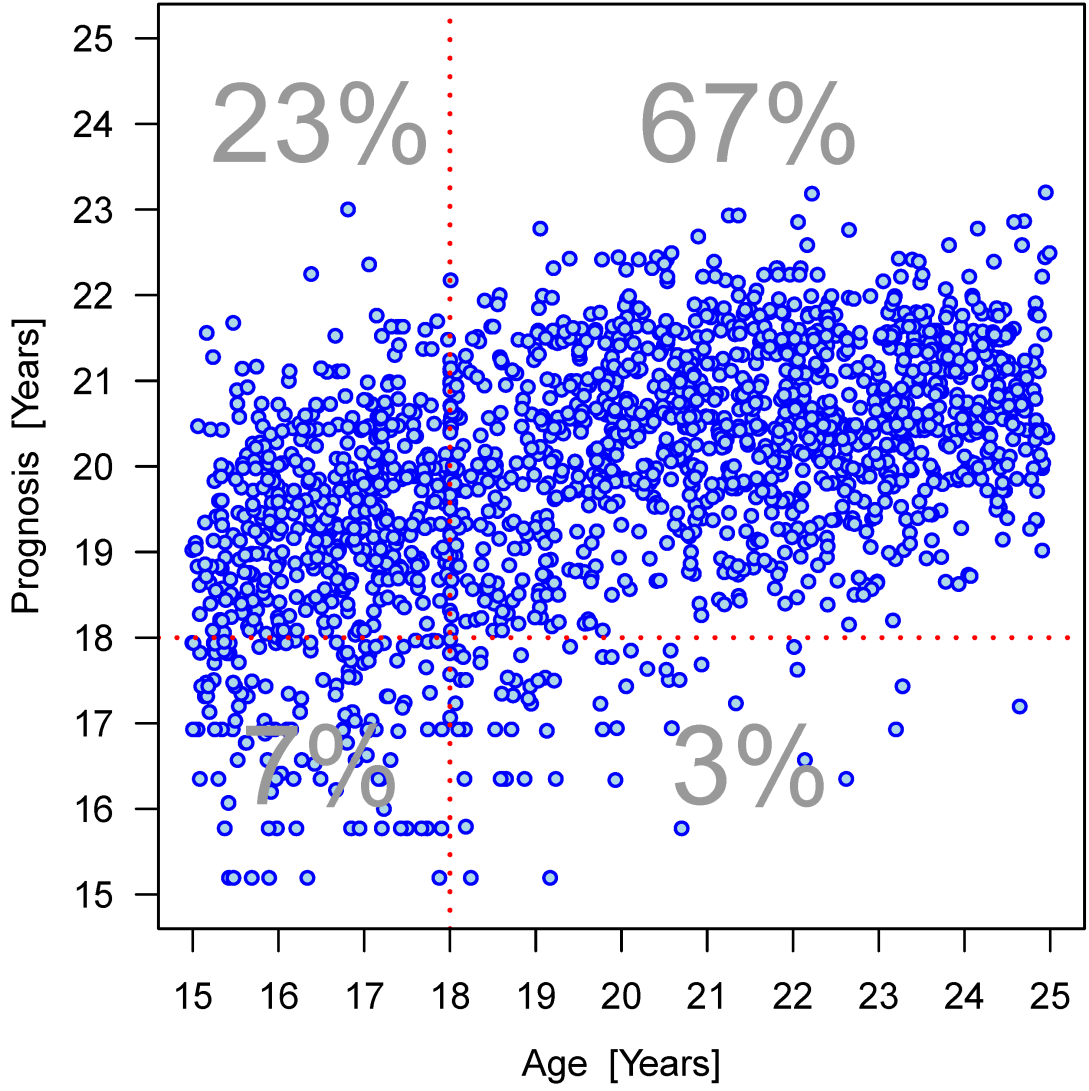
Scattergram of predicted age out of hierarchical models vs. real age for all patients

**Figure 20:**
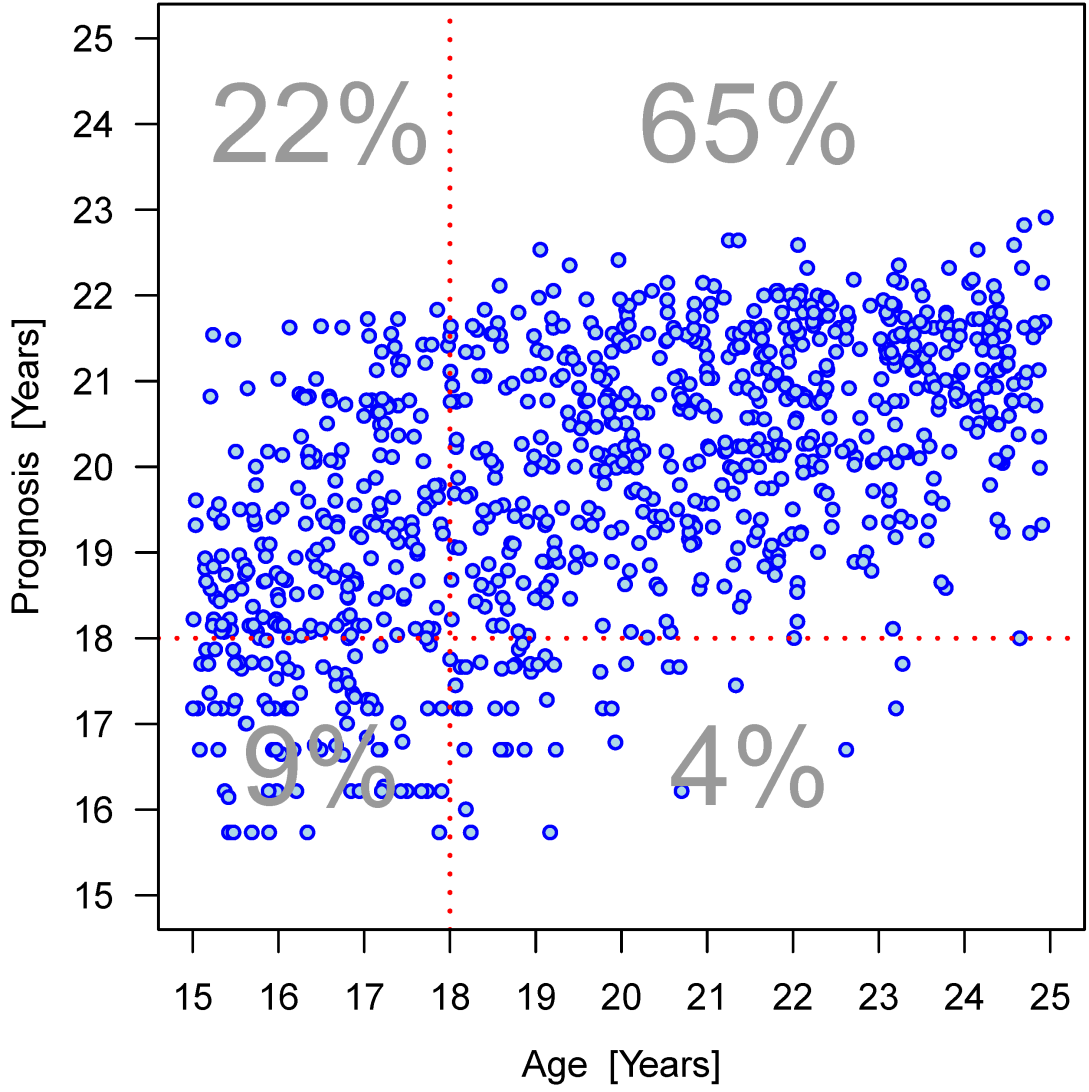
Scattergram of predicted age out of hierarchical models vs. real age for females

**Figure 21:**
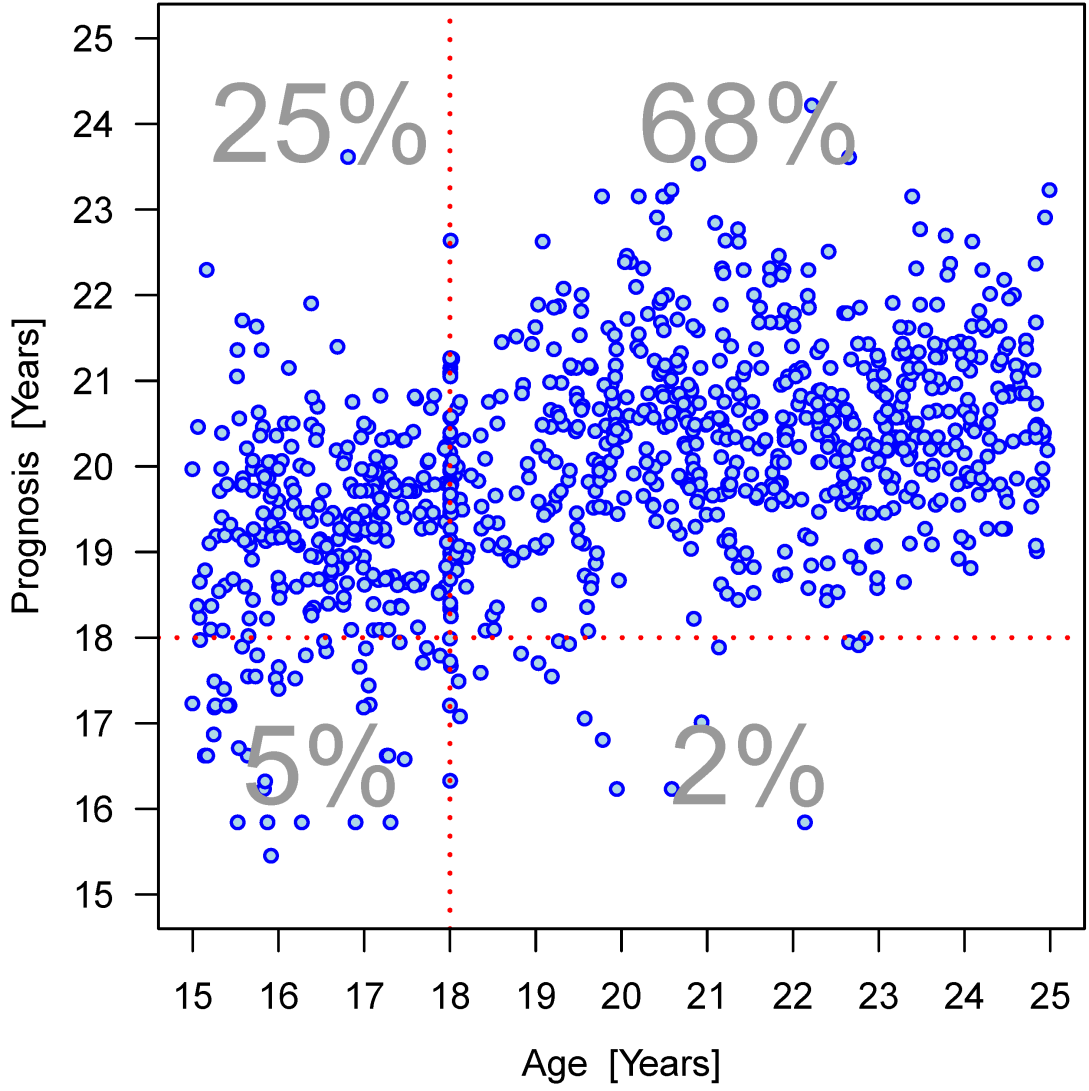
Scattergram of predicted age out of hierarchical models vs. real age for males

Furthermore, the dispersion of the values within an age group can be clearly seen (Figure 15 (Fig. 15), Figure 16 (Fig. 16), Figure 17 (Fig. 17), Figure 18 (Fig. 18), Figure 19 (Fig. 19), Figure 20 (Fig. 20), Figure 21 (Fig. 21)). 

### 2. Multivariate analysis to analyze the development over the time

Hierarchical multivariate analyses were performed to calculate true chronological age depending on the radiographic findings. Initially, all main effects and double interactions were considered and consecutively removed in case of insignificant effects. However, the four main effects were left in the analysis irrespective of level of significance. The effect estimates of the final model were reported as p values with 95% confidence intervals. The following significant influence quantities (p<0.05) were identified related to stage: periodontal space, emergence of wisdom tooth and root development. The alveolar bone loss proved values insufficient for age calculation. An additive factor was calculated for gender (male), periodontal space related to stage, emergence of wisdom tooth related to stage, root development related to stage. Interaction of elongation and root development proved a significant correction of calculation in cases with both variables increasing (Table 11 (Tab. 11)). 

Interaction tests for gender were significant for alveolar bone loss and stage of mineralization, therefore we applied the following stratified model: one for each gender with an individual random term. However, at the first measurement of an individual this random term is of course unknown (Table 12 (Tab. 12)). 

Figure 15 (Fig. 15), Figure 16 (Fig. 16), Figure 17 (Fig. 17) and Figure 18 (Fig. 18) illustrate the predicted values for chronological age plotted against the true chronological age according to the calculations with an analysis of covariance (ANCOVA) that respects the trend in the observed individuals. The graph illustrates the quality of the age prediction. Individuals, who are at least 20 years of age, are reliably predicted as adult (full legal age, 18 years of age or more). On the other hand, the calculation cannot clearly distinguish in a person with a true age of 18 years whether the individual is just below or above this age limit. In fact, as a method of determining frames of expected chronological age the analysis cannot substitute the knowledge of an individual calendrical date of birth. Nevertheless, the calculation allows a more or less precise calculation of individuals who are about 18 years of age. We included an ‘individual term’ in the calculation to specify age determination. Accuracy of age determination is low based on calculation of 4 dental parameters and gender. Relationships within the findings of individual X-ray images were considered in order to improve accuracy of age determination. Initially, all main effects and bilateral interactions were considered followed by successive elimination after proof of insignificance. Table 13 (Tab. 13) and Figure 16 (Fig. 16) demonstrate the improved accuracy of age calculation considering interactions of parameters. Individuals older than 20 years are defined more precisely to be of this age compared to calculations according to the model without consideration of parameter interactions.

We then performed regression analyses to predict the age of the patients based on gender and the four parameters (Table 13 (Tab. 13), Table 14 (Tab. 14), Table 15 (Tab. 15), Figure 19 (Fig. 19), Figure 20 (Fig. 20), Figure 21 (Fig. 21)). Variable selection and search for interaction was identical to the approach described for the hierarchical models. R^2^-values were low suggesting a poor prognostic capacity of the models. 

For further analyses, decision trees were created using the CART (classification and regression tree) algorithm. CART is a recursive partitioning method that explores the structures of a set of data and visualizes decision rules for predicting a categorical outcome [6]. Binary splits (‘nodes’) are made on the predictor variables that best differentiate the outcome variable. We used classification inference trees within the ‘ctree’ function (available in the R package ‘party’), which bases its node splitting on statistical tests [38]. 

**Figure 22:**
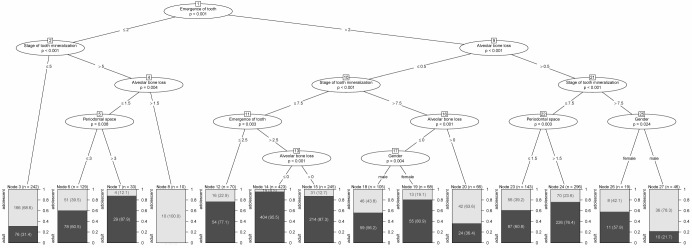
Classification and regression tree (CART) for legal age determination (1,327 patients) versus adolescents (567 patients) based on odontological parameters (whole group)

**Figure 23:**
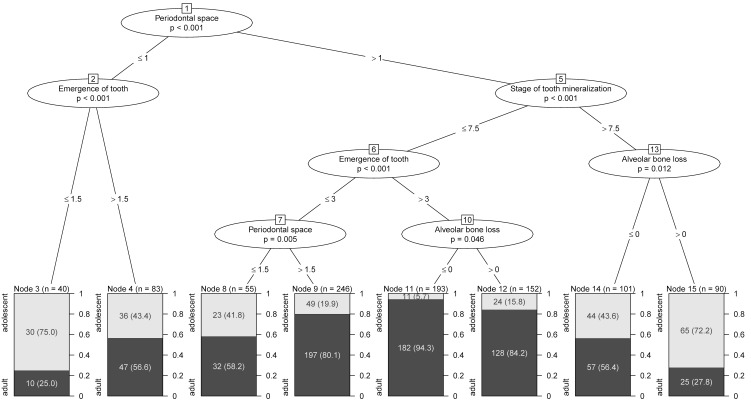
Classification and regression tree (CART) for legal age determination versus adolescents based on odontological parameters (males)

**Figure 24:**
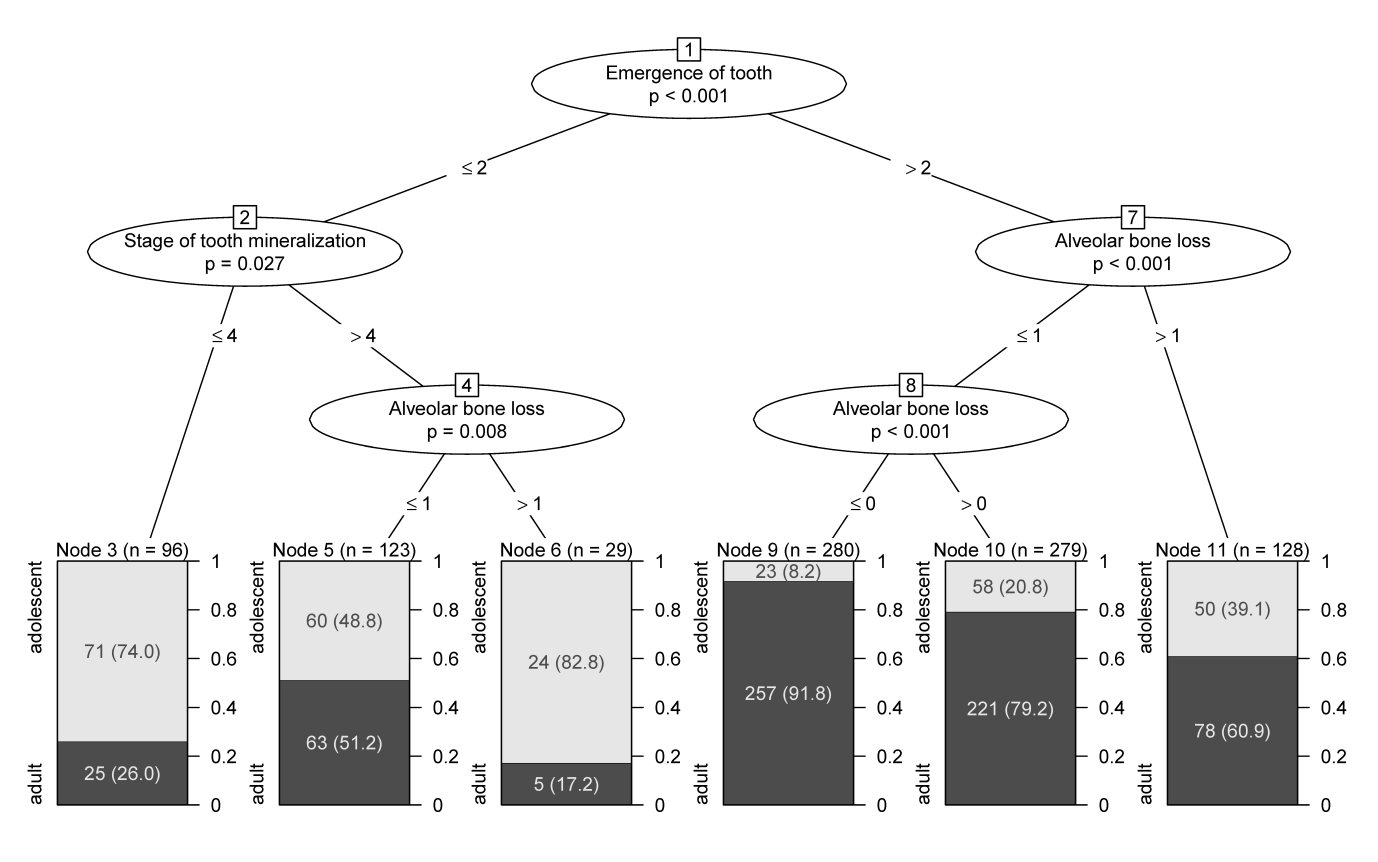
Classification and regression tree (CART) for legal age determination versus adolescents based on odontological parameters (females)

**Figure 25:**
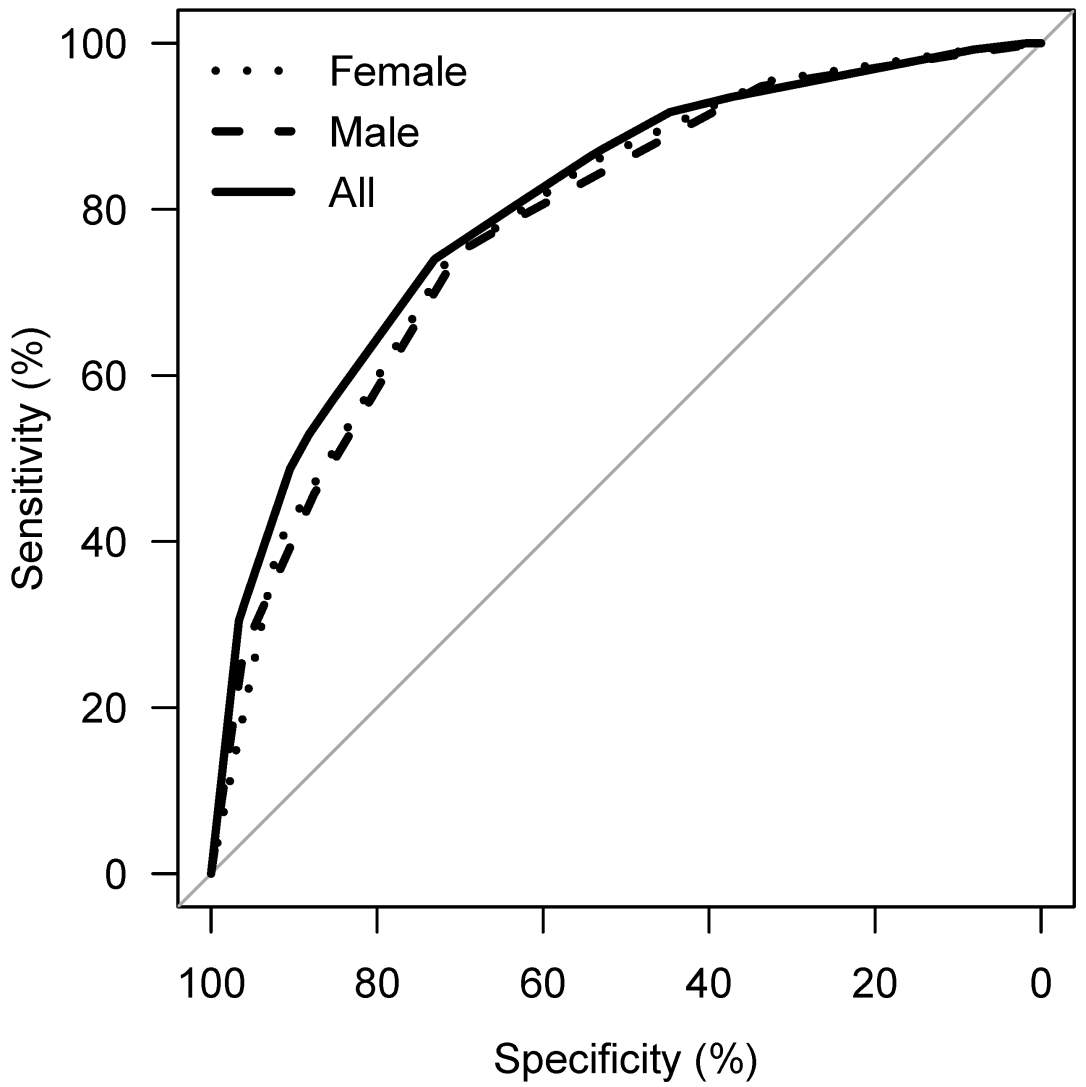
ROC-curves, derived from CART-analyses developed by taking the highest specificity and corresponding sensitivity values to each node

Separate trees were calculated for males, females and for the entire population, respectively (Figure 22 (Fig. 22), Figure 23 (Fig. 23), Figure 24 (Fig. 24), Table 16 (Tab. 16)). For the tree of the entire population three models were calculated according to classification with defining nodes with proportion of patients of legal age >95%, >85% and >75%, respectively. Using this classification, predictions were made and sensitivity and specificity were calculated for each model (Figure 25 (Fig. 25)).

## Discussion

This study provides a clearly and reliably applicable algorithm to calculate the age of 18 years in a group of adolescents and young adults. The odontological age calculation has very obvious limits of measurement accuracy, which have great importance for the medical decision.

Age determination is a frequent requirement in medico-legal practice [89], [90]. Besides the well-known forensic application of dental chart records and dental radiographs in the identification of human corpses [53], [87] the unique spatio-temporal developmental interval of the dentition has attracted broad interest in human sciences, e.g. in the fields of comparative anthropology [10], [17], [25], [26], [48], to detail the human development and its deviations in diseases of internal or external origin [59], to assess dental ages relevant for dental treatment [31], [32], [33], [35], [34], [36], [51] and to correlate certain stages of the dentition with chronological age [5], [8], [40], [107], [103]. For legal purposes, reaching the age of majority is of fundamental importance in many social affairs, including the reservation of consent to medical procedures in individuals younger than 18 years and full capacity of consent in individuals 18 years of age or more. However, this distinction applies only in individuals with sufficient maturity and judgment to understand the intended procedure, i.e. the provided age calculation does not absolve users from the responsibility of evaluating in every case the individual conditions of the patient, to perform a thoroughgoing medical examination and to check the patient’s cognitive ability to be able to understand the personal explanation of the procedure. 

Some stages of wisdom tooth development and further radiological findings of these teeth closely correlate with chronological ages relevant for forensic purposes. Therefore, the (radiological) development stages and the eruption times of wisdom teeth are biometric parameters currently much in use in forensic dentistry [13], [16], [20], [21], [33], [40], [46], [47], [49], [61], [96], [97]. However, the presence of wisdom teeth is not obligate in an individual [65]. In addition to oral inspection [109], [111] further details of the emergence of teeth can be diagnosed on radiographs [76], [79], [110]. Orthopantomography offers an overall view on the teeth of both jaws [16] including topography and developmental stages of third molars [21], [22], and also adjacent bone structures [19]. In addition to radiographic characteristics of the tooth in the narrow sense, the visibility of the periodontal space can be added to the radiographic criteria of the single tooth [51], [71]. Furthermore, the level of the alveolar bone surrounding the lower wisdom tooth [60] was advocated as an age dependent parameter measurable on orthopantomography [23].

### 1. Periodontal space

Olze et al. [71] investigated orthopantomograms of 1198 individuals aged 15 to 25 years with respect to visibility of periodontal *space* of completely mineralized lower wisdom teeth according to the criteria reproduced in Table 2 (Tab. 2). The earliest age where the individuals attained one of the stages was: 17.2 and 17.6 years in females and males (P0), 18.9–20 and 20–20.2 years in females and males (P1), 22.5–23.1 and 22.3 years in females and males (P2), 24.6–25.2 and 25.4–26.2 years in females and males (P3), resp. According to these authors, in individuals reaching P0 the determination of age 18 years is not possible. However, the stages P1–P3 allow the safe determination of an age of 18 years or more, as far as these authors conclude. In particular stages P2 and P3 enables the discrimination of an age of 21 years with high probability. However, these authors also reported that some restraint is called for in determining the age of 21 years based on this item. The results of the presented study allow to conclude an age of at least 18 years to be expected in individuals attaining stage P2–P3 (modified stage 3 and 4 of this study). Nevertheless, the application of this method is technically demanding [14]. Combined with other parameters of wisdom tooth development, the periodontal space is a valuable parameter to field questions from institutions involved in the care for young individuals with unknown chronological age.

### 2. Alveolar bone loss

Periodontal bone loss increases with age but the determination of this item as a single factor to determine the 18^th^ year is not recommended [23]. Most studies on age-dependent periodontal bone loss investigated this factor combined with other dental findings [31], [32], [53], [58], [98]. 

Olze et al. [69] investigated 650 orthopantomograms of German individuals aged 18–30 years. Twenty-five radiographs were collected of each females and males of in every age group. They studied periodontal bone loss of all four second premolars. Only teeth without caries or dental restorations were considered. Bone loss was categorized in four stages. These authors noted a progression of periodontal bone loss with increasing age. In both females and males the increase of median values correlated with increase of stage. However, a considerable variance of values were noted in the medium stages, e.g. the interquartile distances of stages 1 and 2 were 3 to 8 years. Starting with stage 1, all median values of all teeth of this study and in both sexes were at least 21 years. Therefore, a periodontal bone loss of this stage is likely to occur in individuals aged 21 years. Lower quartile of 21 years were regularly associated with stage 2, i.e. 75% of individuals with progressive periodontal disease were at least 21 years of age. Stage 3 was found earliest in men aged 25 years and was a rare finding (3.7%). On the other hand, stage 3 was occasionally identified in females 20 years of age [69]. 

The interpretation of own results has to point out that there is no close relationship between age and alveolar bone loss in the wisdom tooth region. The main reason for the lack of consistency between these 2 parameters is probably the transient bone loss occurring during emergence of third molars. The emergence of wisdom teeth into the oral cavity is inevitably associated with development of pericoronal niches allowing plaque accumulation and consecutive inflammatory osseous disintegration [60]. These extrinsic factors superimpose bone remodeling and thus bone loss is no reliable factor to calculate the target age of this study.

### 3. Emergence of wisdom tooth

The scientific literature on wisdom tooth emergence is based on one side on radiologic studies on tooth development [15] and on the other side on clinical studies with a focus on the clinical process of tooth eruption into the oral cavity [111], [109], or the combination of investigating techniques [16], [35]. There is a large body of literature addressing the applicability of dental parameters to define the individual chronological age [87]. This report only touches upon some relevant reports in the context of the applied method.

Emergence of teeth and in particular the emergence of wisdom teeth is frequently the only valid odontologic source of forensic age estimation in children and adolescents [18]. This emphasis on the impact of wisdom tooth developmental stages and age assessment is even more evident in cases where radiographic investigations are not applicable. However, oral inspection of teeth without current radiological images of the jaws has important limitation. There are reports on noteworthy differences of third molar eruption related to ethnicity [10], [65], [79], [96]. 

Haavikko [34], [35] performed a radiological analysis on tooth eruption and mineralization using orthopantomograms of 615 male and 547 female Finnish individuals. Wisdom teeth had a special status, because the findings of these teeth were recorded only with respect to the penetration through the alveolar bone. According to this definition of tooth emergence wisdom teeth passed through the bone at the age of 17.2 and 18.1 years, resp. (Standard deviation 3.9 to 6.3 years, accelerated wisdom tooth eruption in females compared to males was 0.7 years). Maxillary wisdom teeth eruption preceded the eruption of lower wisdom teeth by 0.1 to 0.3 years (mean values). About 50–60% of the individuals comprising the oldest age group showed wisdom teeth emerging into the oral cavity.

Gleiser and Hunt [28] and Garn et al. [25], [26] defined alveolar emergence as the eruption of a tooth cusp or the complete occlusal tooth surface above the level of the alveolar process. Most authors agree that combination of the findings of both sides of the jaw are justified because developmental stages of teeth deviate not significantly between sides of one jaw. 

Clinical studies are beyond the scope of this report. However, it has to be noted that third molar emergence into the oral cavity appears to imply considerable ethnic variations. While the third molar eruption into the oral cavity usually does not take place prior to the 17^th^ year of life in European populations [65], other authors, e.g. Chagula [10], Otuyemi et al. [79] and Shouri [96] report on earlier wisdom tooth eruption in their populations. Several studies of Olze et al. [73], [77], [78], [72] performed inter-ethnic comparisons to define more precisely the probable effect of ethnicity on wisdom tooth development and emergence. They studied 2,482 orthopantomograms of 660 Germans, 1,300 Japanese and 519 black South Africans with known birth dates. The investigation was performed by one of the authors in all cases. In this study, different stages of tooth emergence were defined: stage A (occlusal surface covered with alveolar bone in the direction of estimated emergence), stage B (penetration of alveolar bone), stage C (gingival eruption), and stage D (tooth grown into occlusion). The German population hold an intermediate position to reach a particular stage of wisdom tooth emergence. Statistically significant differences between the populations were found in females of stages A, B and C. South African females reached these stages about 1.6–1.8 years prior to German females. On the other hand, Japanese females were about 0.9–3.3 years older than German females when they reached these stages. In males statistically significant differences of wisdom tooth emergence were found in stages A and B. South African black male were about 3.0–3.2 years younger than German males. However, Japanese males were about 3.1–4.2 years older than South African males when they reached stages A and B. This study describes in detail ethnic differences of stages of wisdom tooth eruption, thereby supporting our hypothesis to calculate chronological age by means of combined dental findings of wisdom teeth on standard dental radiographs. Indeed, an earlier study on the relation of wisdom teeth’ crown position to the occlusal plane proved no predictive data to calculate the age of 18 years [110].

In our study, females’ wisdom tooth emergence was in advance compared to males in early stages of the process and found to be delayed in the final stages. No side differences of tooth emergence were noted, as in previous studies. However, the clinical aspect of eruption into the oral cavity was beyond the scope of this study, as was the data acquisition of the ethnic background of patients treated in a large city of Germany.

### 4. Root development and tooth mineralization

Different staging system were proposed to assess the mineralization of dental hard tissues that were also applied for correlation studies of chronological age. These differences between staging systems allow no direct comparison of results. Furthermore, the composition of the reference populations as the basis for predictive values in the calculation of chronological age differed markedly [36], [80]. For example, staging system were published by Gleiser and Hunt [28], Nolla [67], Haavikko [34], Demirjian et al. [15], Gustafson and Koch [33], Harris and Nortje [37], Kullman et al. [49], and Köhler et al. [47], to mention just a few of the researchers included in age determination studies. From a scientific point of view, the reliability of staging systems to predict the chronological age is of uttermost importance. Several of the above mentioned classifications rely on the application of numerous stages of tooth development that appear to allow a fine graduation of dental developmental stages and consequently fine adjustment to chronological age but also facilitate subjective errors of measurement [14]. Demirjian et al. [15] published a staging system of tooth development based on four stages each of crown and root development. These eight stages are defined according to radiological changes of the tooth’s shape on plain radiographs and are independent from speculative length estimations that are prone to errors in measurement due to radiographic projections. Furthermore, a numerical code of stages was avoided in order to exclude the assumption the stages were defined by equal time intervals. This staging system is currently widely applied in the fields of forensic dentistry [87], [114]. A recent analysis dedicated to compare the validity and practicability of radiological staging systems of tooth development provided evidence for the superiority of the Demirjian method [48], [56], [68]. However, the correlation between the radiological assessed developmental stage of a wisdom tooth and the developmental stage of the inspected extracted tooth is less than 100% [17]. On the other hand, the time frame is large concerning the eruption of the wisdom tooth into the oral cavity [9], [39].

In the anthropological, dental and legal literature it is generally accepted that the apical root closure takes place during the age of 20 to 23 years [43], [46], [62], [77]. The probability of a completely developed wisdom tooth prior to the age of 18 years is very small, but cannot generally be excluded [33], [46], [62]. This assessment was confirmed in the present study.

Impaction of wisdom teeth had no impact on the time frame on the expected completion of wisdom tooth mineralization [21]. However, this finding was not confirmed in studies on third molar mineralization in black Africans: in this population the impacted wisdom teeth mineralized more slowly than non-impacted third molars [78]. 

A sexual dimorphism is an expected finding in tooth development [35], [34], [54]. However, this finding is not supported by some researchers [13]. In the present study, gender had an impact on the radiologically assessed root formation during the chosen study period. Therefore age calculations have to consider gender.

## Conclusion

The application of dental radiography in the context of age estimation in humans, with special reference to determine ages that are crucial for the right of informed self-determination, can be structurally improved by a combined analysis of lower wisdom teeth findings, as shown in this study. The analysis provides a formula to calculate the probability of a chronological age 18 years or more in a given individual that is previously estimated to be in the age period of 15 to 24 years. The method has, however obvious and severe limitations. Therefore, odontological age estimation by determining lower wisdom tooth growth stages should be incorporated with care into the general medical examination in order to assess chronological age. The calculation may be of importance where there is a need to clarify whether the person can decide on an intended medical intervention by itself or if there is need for further legal support. In particular, decisions in planning elective surgical interventions may have some benefit from the provided data. Nevertheless, further studies are encouraged to investigate the applicability of this age calculation in different ethnic groups.

## Notes

### Competing interests

The authors declare that they have no competing interests.

### Authorship

The authors REF and KS contributed equally to this publication.

## Figures and Tables

**Table 1:**
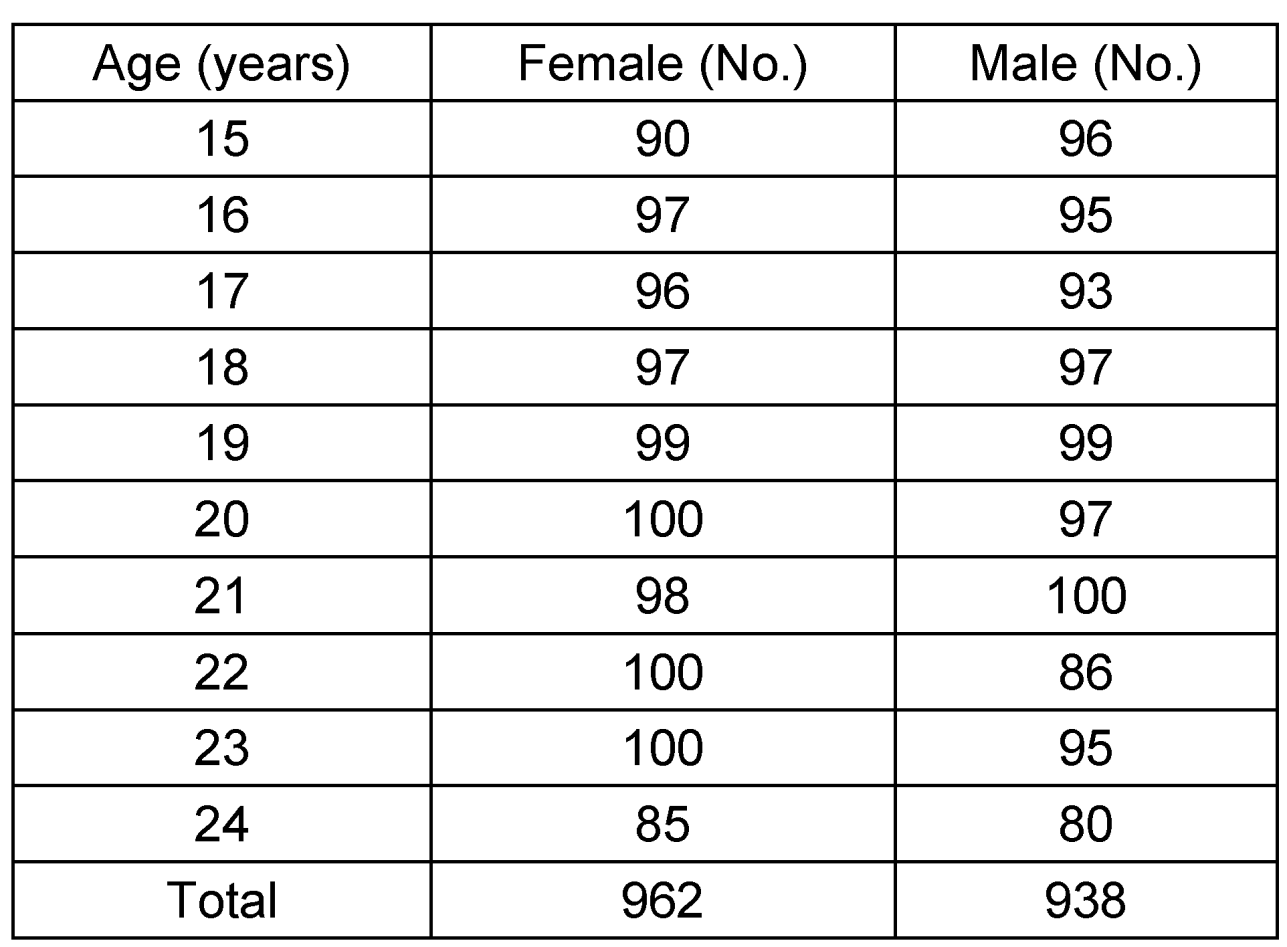
Age and gender of study group (N=1,900)

**Table 2:**
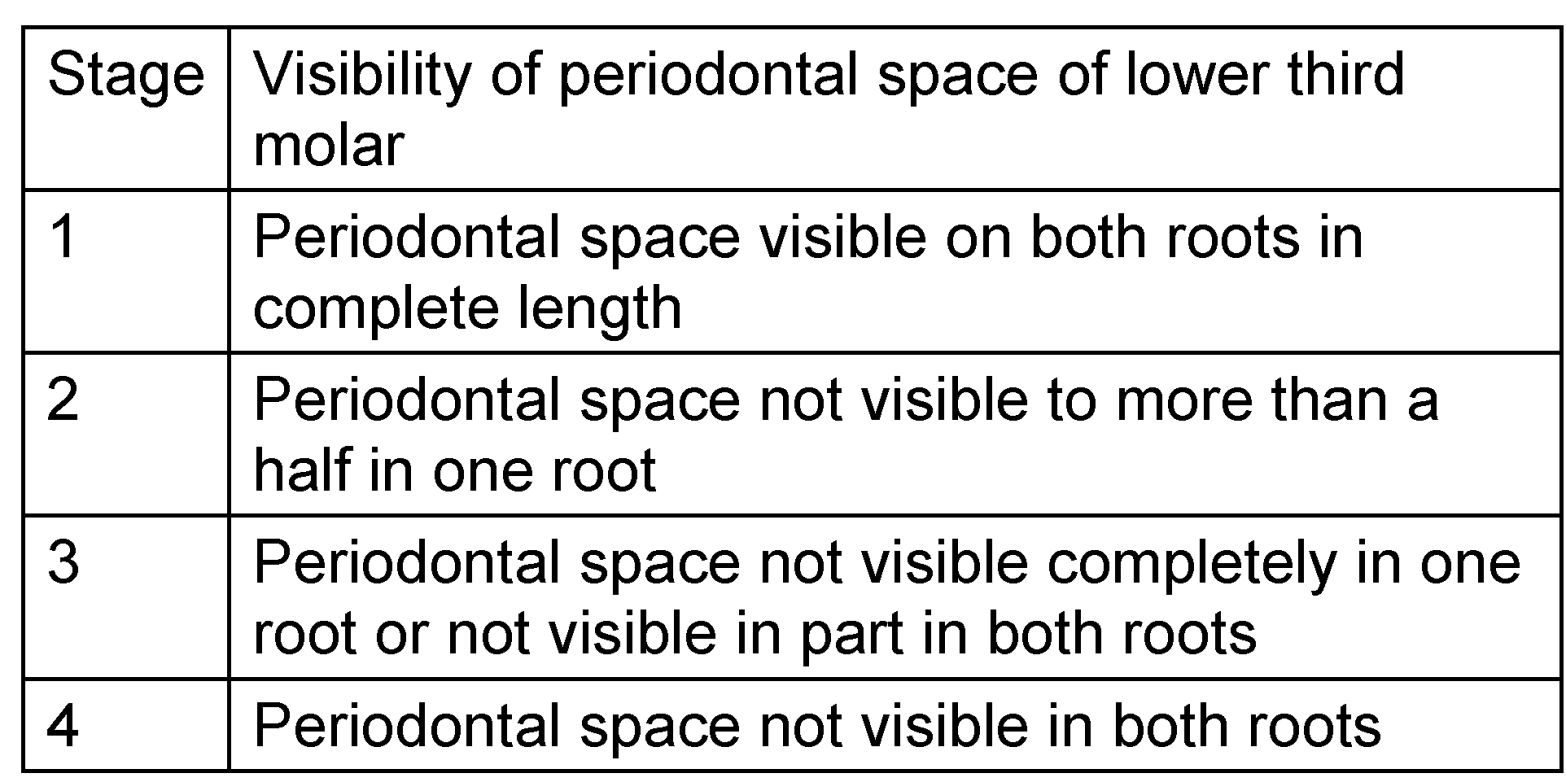
Stage grouping of the feature ‘periodontal space’ on orthopantomograms [75]

**Table 3:**
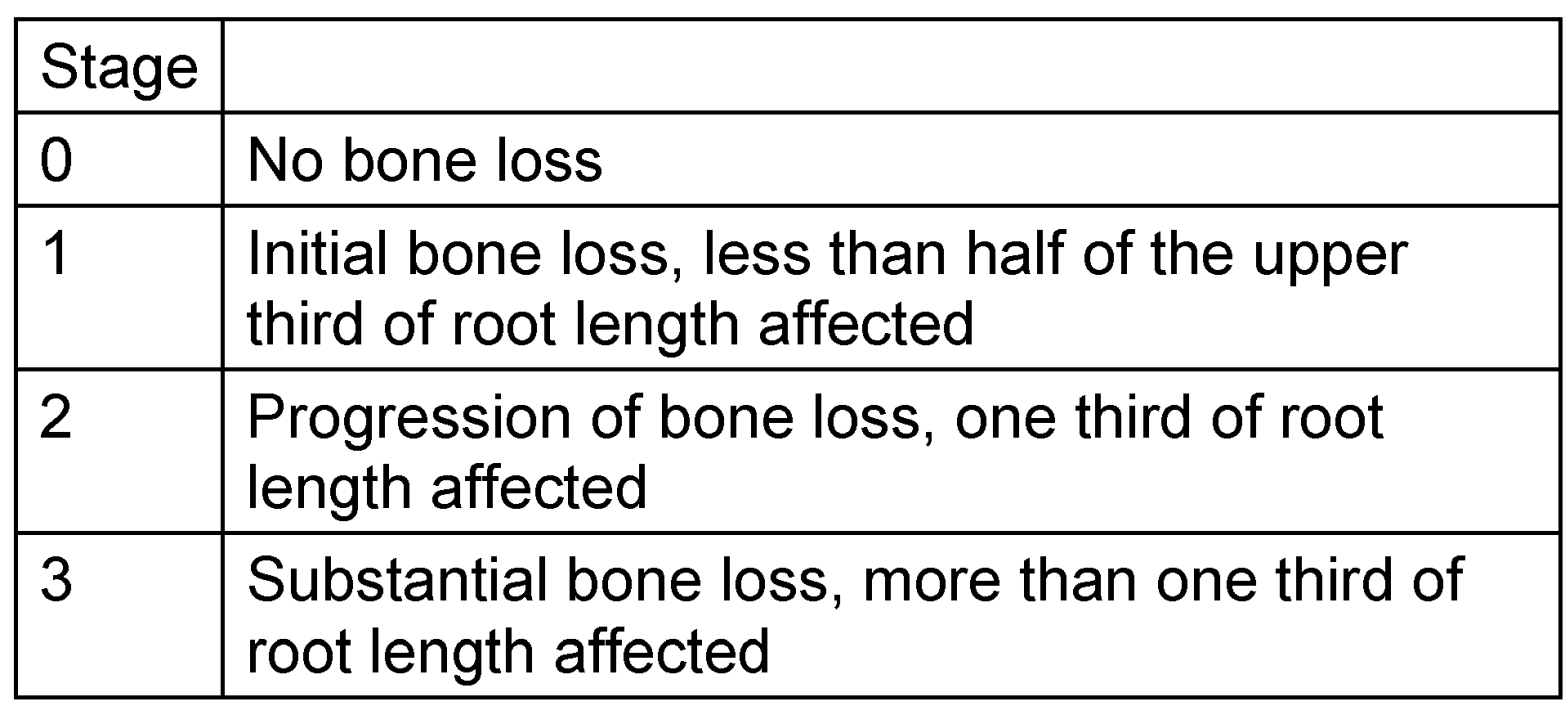
Stage grouping of periodontal bone loss according to Olze et al. [69]: Instead on premolars the classification was applied to the alveolar process of second and third lower molars.

**Table 4:**
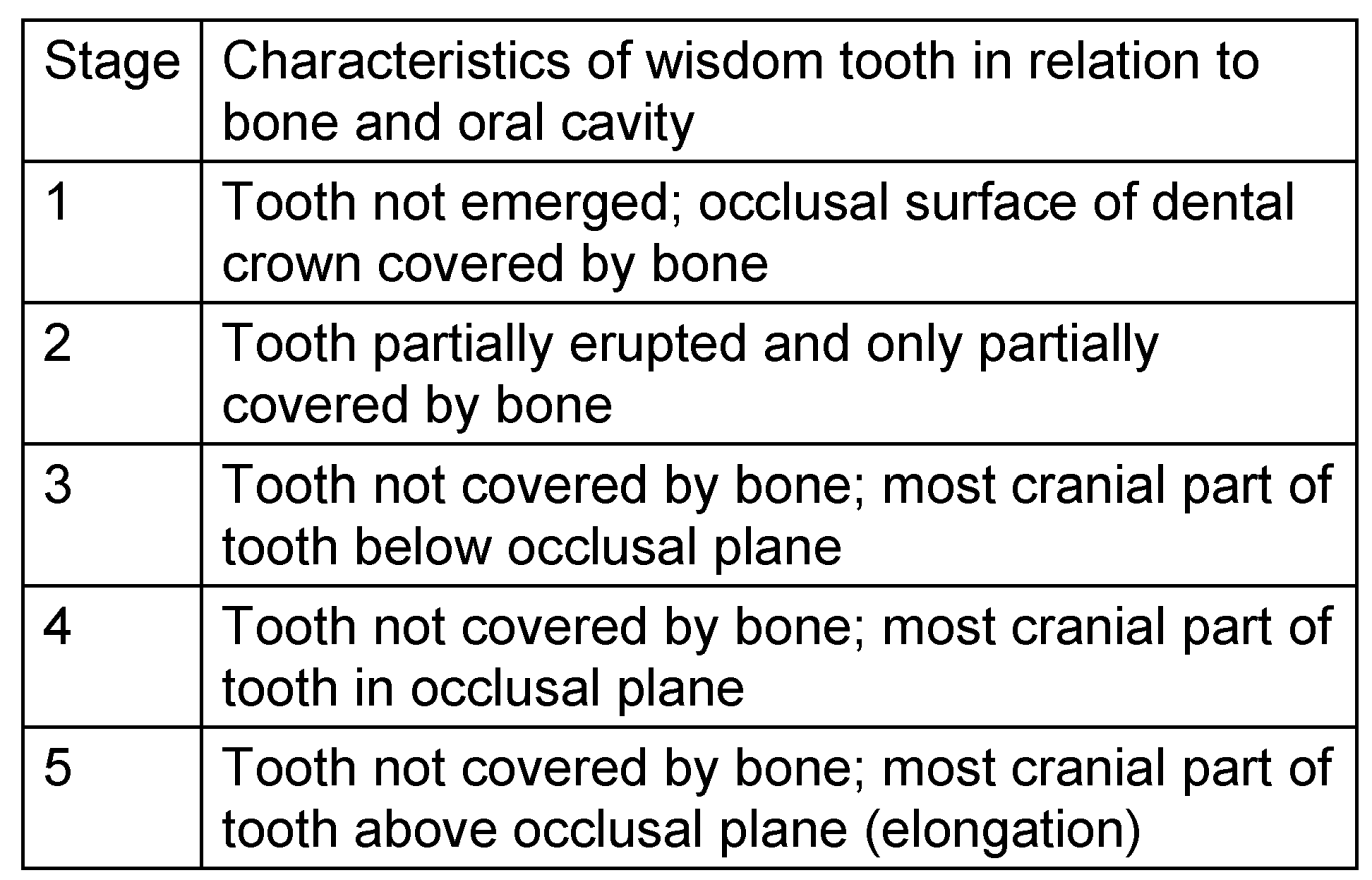
Stage grouping of wisdom tooth eruption [78]

**Table 5:**
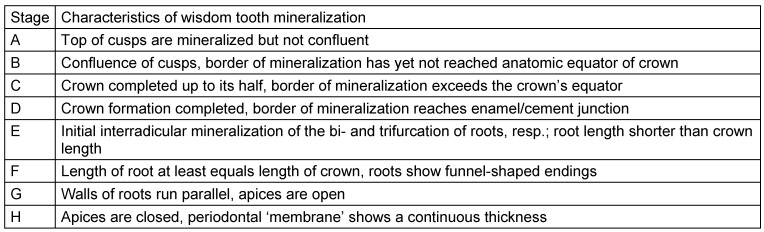
Stages of wisdom tooth mineralization [15]

**Table 6:**
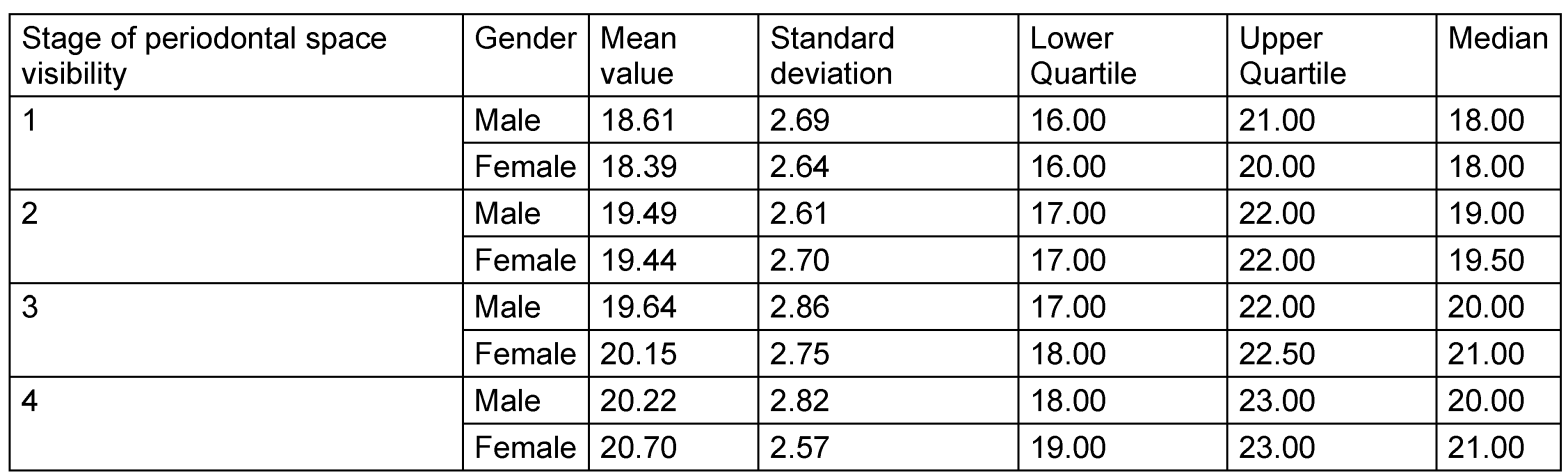
Visibility of periodontal space on orthopantomograms of the lower wisdom teeth (Tooth No. 38 and 48) staged according to Olze et al. [75] and with respect to gender

**Table 7:**
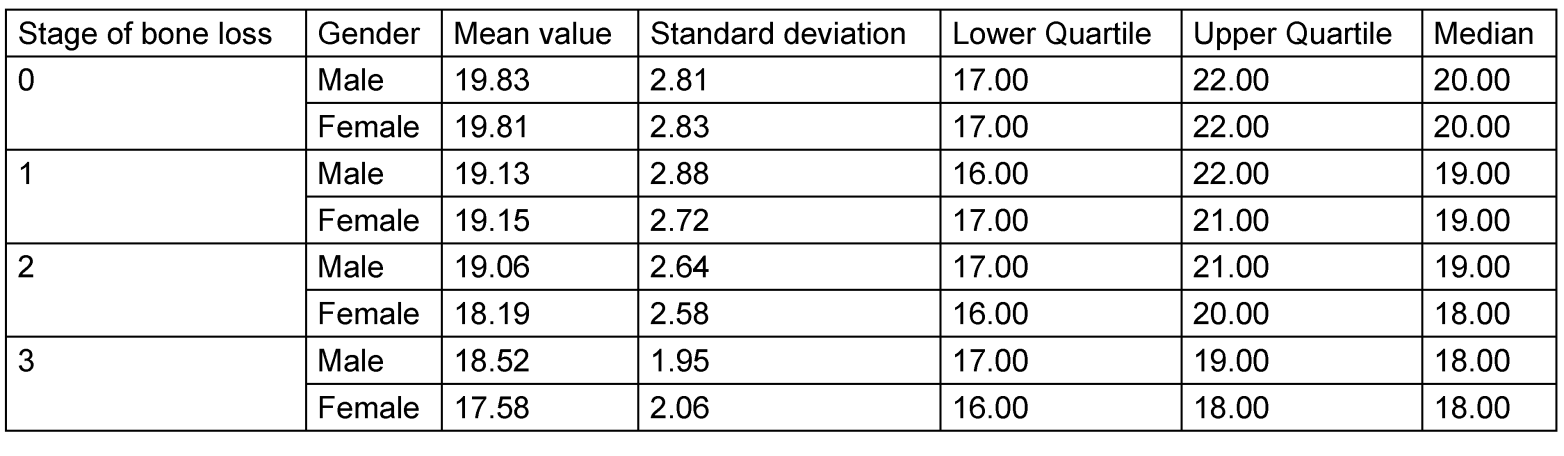
Alveolar bone loss of lower wisdom teeth (No. 38 and 48)

**Table 8:**
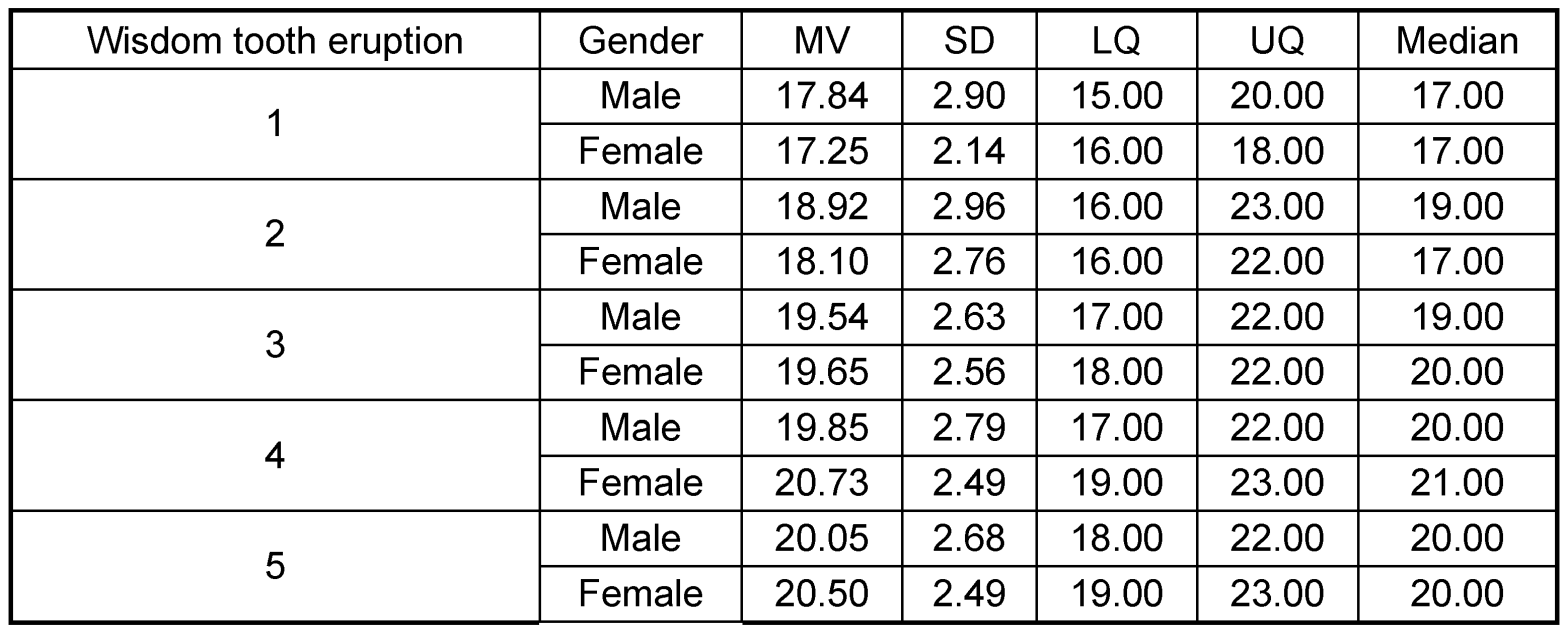
Lower wisdom tooth emergence (teeth 38 and 48) MV = Mean Value, SD = Standard Deviation, LQ = Lower Quartile, UQ = Upper Quartile

**Table 9:**
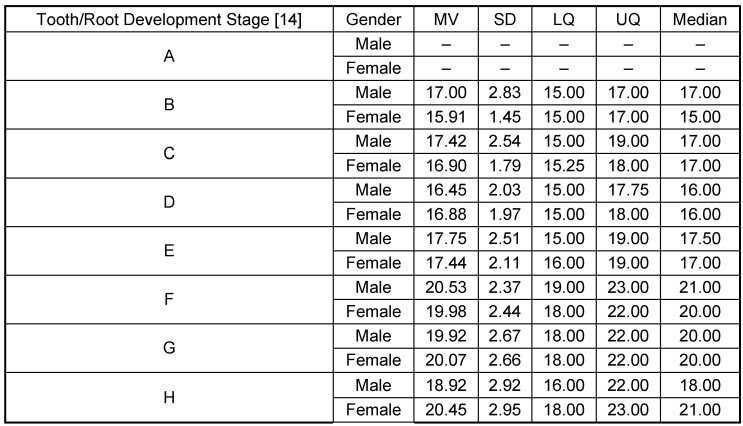
Root development (mineralization) of lower wisdom tooth (teeth 38 and 48). MV = Mean Value, SD = standard deviation, LQ = Lower Quartile, UQ = Upper Quartile. Fig. 11 illustrates the values of Table 9.

**Table 10:**
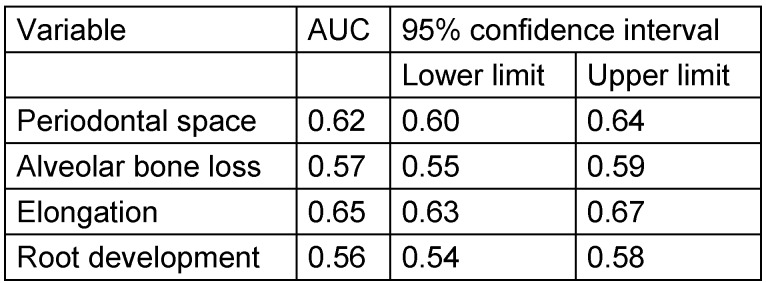
ROC measures related to attained majority (18 years) depending on radiographic findings of wisdom teeth on orthopantomogram, total group (AUC = area under the curve, ROC = receiver operating characteristics)

**Table 11:**
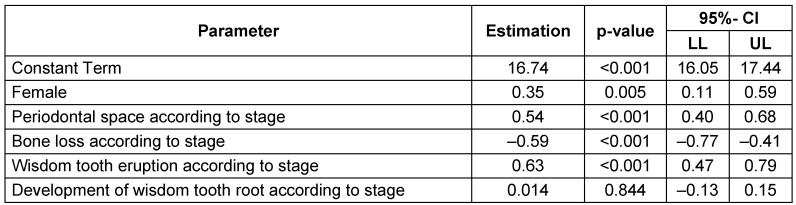
Multivariate regression model with main effects on the target variable ‘age’ CI = confidence interval, LL = lower limit, UL = upper limit. R^2^=0.157

**Table 12:**
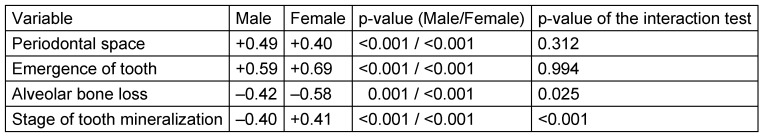
Interaction tests

**Table 13:**
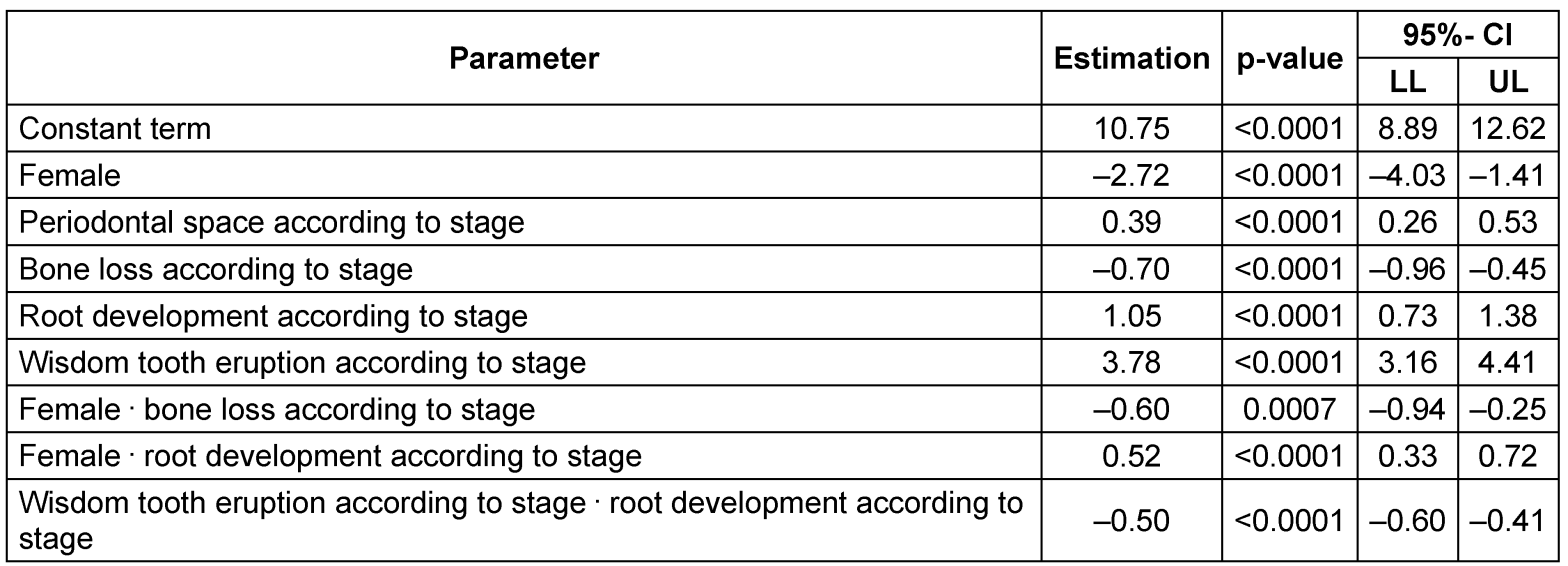
Multivariate regression model considering interactions of parameters on the target variable ‘age’ CI = confidence interval, LL = lower limit, UL = upper limit. R^2^=0.23

**Table 14:**
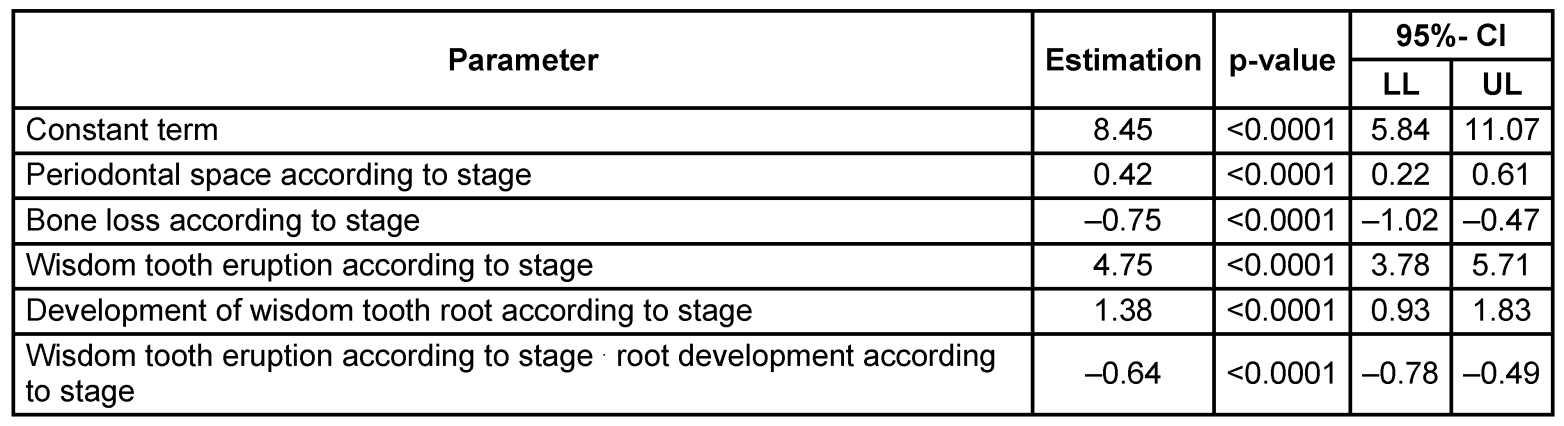
Multivariate regression model for males considering interactions of parameters on the target variable ‘age’ CI = confidence interval, LL = lower limit, UL = upper limit R^2^=0.31

**Table 15:**
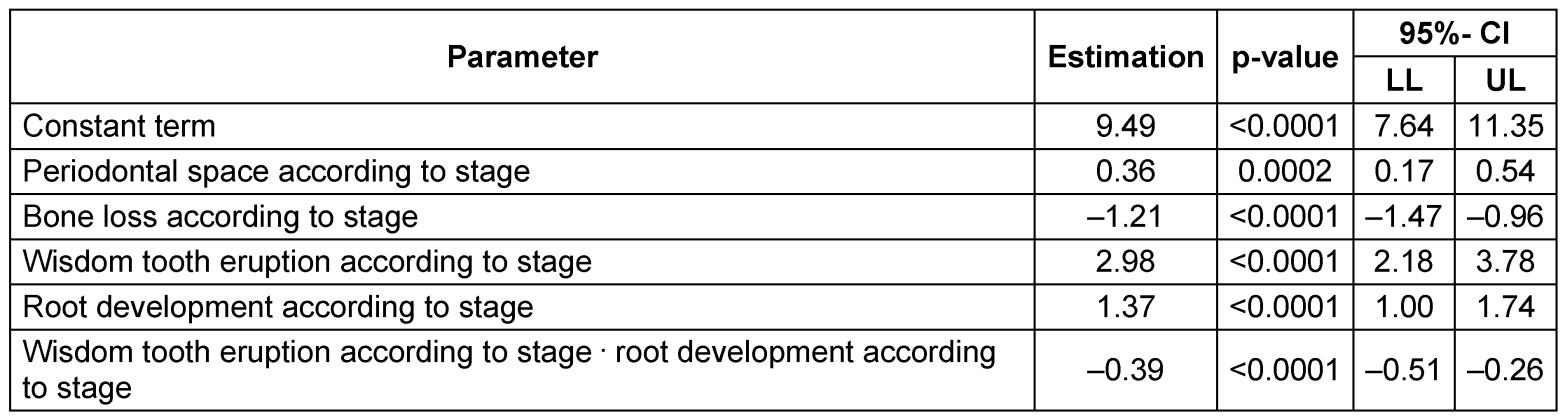
Multivariate regression model for females considering interactions of parameters on the target variable ‘age’ CI = confidence interval, LL = lower limit, UL = upper limit. R^2^=0.16

**Table 16:**
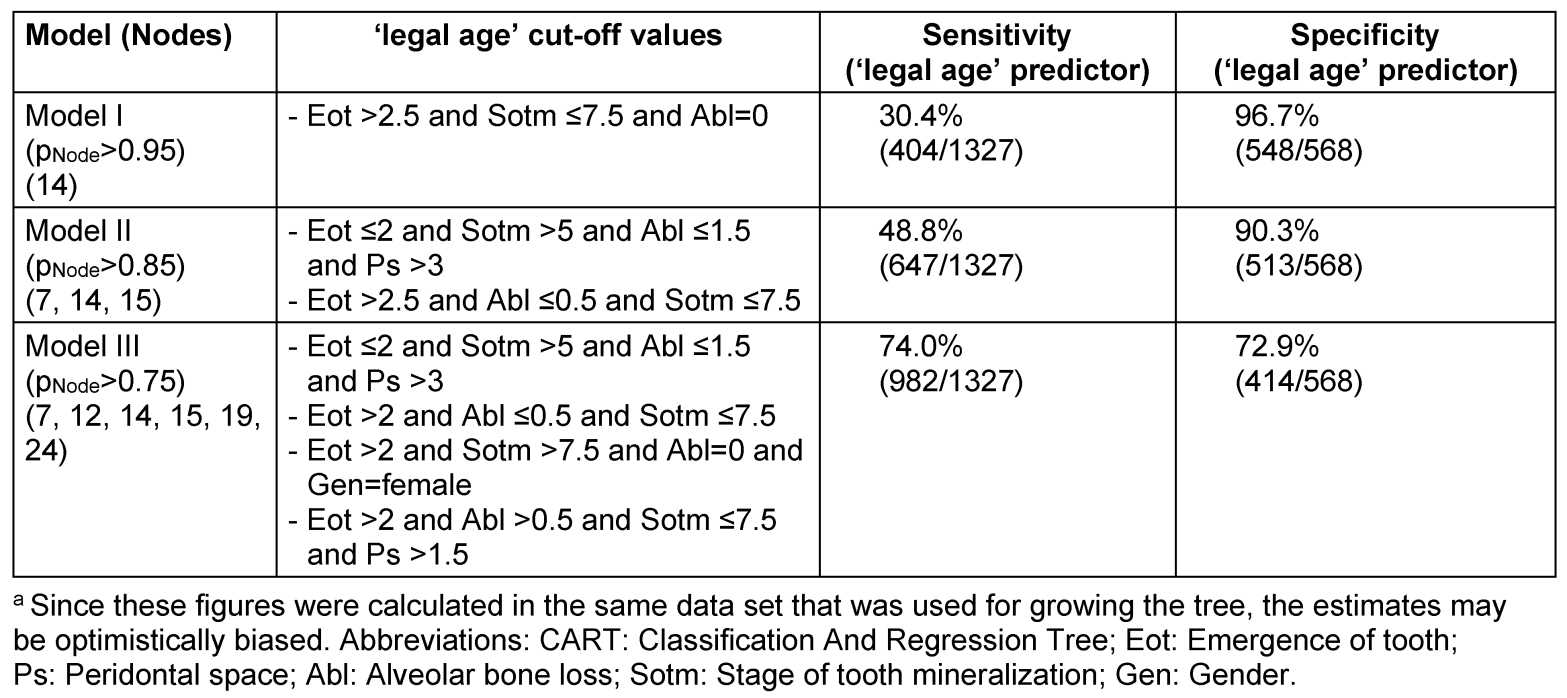
Sensitivity and specificity of different 'legal age' predictors derived from CART ^a^
